# Hydrogen‐Generating Magnesium Alloy Seed Strand Sensitizes Solid Tumors to Iodine‐125 Brachytherapy

**DOI:** 10.1002/advs.202412263

**Published:** 2024-12-10

**Authors:** Pan Hu, Letao Lin, Guanyu Chen, Dengyao Liu, Huanqing Guo, Meigui Xiao, Zhihui Zhong, Guang Yang, Bingchen Xu, Dongcun Huang, Sheng Peng, Yong Li, Yanling Zhang, Tao Huang, Fujun Zhang

**Affiliations:** ^1^ Department of Minimally Invasive Intervention State Key Laboratory of Oncology in South China Guangdong Provincial Clinical Research Center for Cancer Sun Yat‐sen University Cancer Center Guangzhou 510060 P. R. China; ^2^ Zhuhai Interventional Medical Center Zhuhai Precision Medical Center Zhuhai People's Hospital Zhuhai Hospital Affiliated with Jinan University Zhuhai 519000 P. R. China; ^3^ School of Laboratory Medicine and Biotechnology Southern Medical University Guangzhou 510515 P. R. China

**Keywords:** brachytherapy, hydrogen, iodine‐125 seeds, magnesium alloy, radiosensitivity

## Abstract

Radioactive iodine‐125 (^125^I) seed implantation, a brachytherapy technique, effectively kills tumor cells via X‐rays and gamma rays, serving as an alternative therapeutic option following the failure of frontline treatments for various solid tumors. However, tumor radioresistance limits its efficacy. Hydrogen gas has anticancer properties and can enhance the efficacy of immunotherapy. However, its role in radiotherapy sensitization has rarely been reported. Many current hydrogen delivery methods involve hydrogen‐generating nanomaterials, such as magnesium‐based nanomaterials. This study introduces an AZ31 magnesium alloy ^125^I seed strand (termed AMASS) with pH‐dependent slow‐release hydrogen characteristics and excellent mechanical properties. AMASS can be implanted into tumors via minimally invasive surgery, releasing hydrogen around the ^125^I seeds. In vitro experiments showed that hydrogen from AMASS degradation significantly inhibited tumor proliferation, increased apoptosis, disrupted redox homeostasis and mitochondrial membrane potential, reduced adenosine triphosphate levels, and induced DNA damage due to ^125^I radiation. In mouse xenograft and rabbit liver tumor models, hydrogen from AMASS showed superior therapeutic effects compared with ^125^I seeds alone, with no noticeable side effects. In addition, AMASS has a uniform radiation dose distribution and simple implantation. Therefore, hydrogen from AMASS enhanced ^125^I seed efficacy, supporting the further promotion and application of ^125^I seed implantation in cancer therapy.

## Graphical abstract

This study introduces AMASS, an AZ31 magnesium alloy ^125^I seed strand that releases hydrogen gas to enhance the efficacy of radioactive ^125^I seed implantation in treating solid tumors. In vitro and in vivo experiments demonstrate that hydrogen from AMASS improves tumor response by increasing apoptosis, inducing DNA damage, and enhancing radiosensitivity, with minimal side effects and a uniform radiation dose distribution.

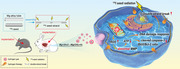

## 1 Introduction

Solid tumors, which are masses detectable through various diagnostic methods, constitute a significant portion of the global cancer burden. According to recent reports, there are ≈19.3 million new cancer cases worldwide and ≈10 million cancer‐related deaths, the vast majority of which are due to solid tumors.^[^ ^1^ ^]^ Despite the numerous treatment options available for solid tumors, such as radiofrequency ablation, stereotactic body radiation therapy, and transarterial chemoembolization, prolonged treatment duration may lead to the development of resistance to standard frontline therapies. One approach for subsequent treatment is brachytherapy, which involves the implantation of radioactive iodine‐125 (^125^I) seeds directly into the tumor tissue under imaging guidance or direct visualization.^[^ ^2^ ^] 125^I seeds emit low‐energy X‐rays and gamma rays with a sustained steep dose gradient and low dose rate, making them particularly effective in targeting tumors while minimizing damage to the surrounding normal tissues.^[^ ^3^ ^]^ This method has been successfully applied to treat various solid tumors, including hepatocellular carcinoma, colorectal cancer, cervical cancer, pancreatic cancer, and prostate cancer.^[^ ^4^, ^5^, ^6^, ^7^, ^8^ ^]^ The implantation of ^125^I seeds represents one option for salvage therapy, particularly suitable for liver metastases in challenging locations (diaphragm, gallbladder, hepatic hilum, or intestines) or those that have failed other treatment modalities. Despite these successes, some patients still experience poor outcomes due to tumor cell radioresistance and irregular seed placement, which can result in uneven radiation doses and tumor recurrence.

The primary cause of brachytherapy failure is the development of radioresistance in tumor cells, which generally stems from changes in two classical pathways. First, the DNA damage response (DDR) is a critical factor. Ionizing radiation causes direct and indirect DNA damage. Direct damage occurs when radiation directly affects DNA molecules, whereas indirect damage results from the radiation‐induced production of reactive oxygen species (ROS) that damage the DNA.^[^ ^9^ ^]^ When DNA damage occurs, cells initiate the DDR, which involves damage recognition, signal transduction, and DNA repair.^[^ ^10^ ^]^ Radioresistance occurs if the DDR capability of tumor cells exceeds the extent of DNA damage. Thus, increasing DNA damage or inhibiting the DDR pathway is a feasible approach for radiosensitization. Escape from apoptosis is another significant pathway that contributes to radioresistance. When DNA repair fails, tumor cells can evade apoptosis, often through upregulation of the anti‐apoptotic protein Bcl‐2 and downregulation of the pro‐apoptotic protein BAX.^[^ ^11^, ^12^ ^]^ Targeting the BAX/Bcl‐2 pathway has been shown to enhance tumor radiosensitivity. Therefore, identifying a radiosensitizer that inhibits the DDR pathway and promotes apoptosis in tumor cells could effectively overcome radioresistance to brachytherapy.

Hydrogen gas, a therapeutic medical gas, has been demonstrated to have antitumor properties. Research dating back to 1975 showed that high‐pressure hydrogen can promote the regression of skin squamous cell carcinoma.^[^ ^13^ ^]^ Because of the extremely low solubility of hydrogen in water, conventional hydrogen delivery methods, such as drinking hydrogen‐rich water, are insufficient to achieve therapeutically effective hydrogen concentrations within tumors.^[^ ^14^ ^]^ With the advancements in materials science, recent studies have increasingly focused on hydrogen‐generating or hydrogen‐carrying nanoparticles that can be administered intravenously to exert therapeutic effects. This material‐based hydrogen delivery approach leverages the enhanced permeability and retention (EPR) effect of solid tumors and the high hydrogen‐loading capacity of nanoparticles, effectively increasing the intratumoral hydrogen concentration.^[^ ^15^, ^16^ ^]^ Thus, developments in materials science have significantly advanced the application of hydrogen in the treatment of solid tumors. Moreover, the small molecular size of hydrogen allows it to rapidly diffuse into tissues and cells where it can selectively reduce toxic ROS and modulate mitochondrial function, thereby inhibiting tumor growth.^[^ ^17^ ^]^

We aimed to address the radioresistance of solid tumors to ^125^I seed therapy using hydrogen‐generating materials. The primary feature of this material is its ability to slowly release hydrogen gas, corresponding to an ≈1‐month efficacy period for ^125^I seed brachytherapy. Additionally, because this material is implanted into tumors via an interventional approach, it must exhibit good biocompatibility. Finally, this material should facilitate co‐implantation with ^125^I seeds, provide structural support, and ensure dose uniformity. Therefore, we chose magnesium alloy tubes as radiosensitizers for brachytherapy to treat tumors. In recent years, biodegradable Mg alloys have gained attention owing to their potential for clinical application.^[^ ^18^, ^19^ ^]^ Magnesium, known for its castability, tensile strength, and availability, has been used in various medical implants, including vascular and bone scaffolds.^[^ ^20^, ^21^ ^]^ Studies have shown that magnesium alloys can also play a significant role in the treatment of solid tumors^[^ ^22^, ^23^ ^]^ because they can react with water to produce hydrogen gas (H_2_), magnesium ions (Mg^2+^), and hydroxide ions (OH^−^).

**Figure 1:**
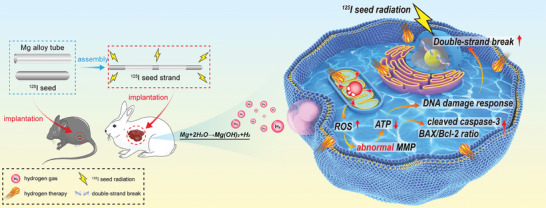
Schematic diagram of the assembly and potential application of the novel AZ31 magnesium alloy seed strand.

In this study, we aimed to validate the therapeutic potential of an AZ31 magnesium alloy seed strand (AMASS) (**Figure** **1**). Our primary objective was to determine whether the hydrogen gas produced by the degradation of AMASS enhances tumor radiosensitivity and to elucidate the underlying mechanisms. AMASS was created by alternately connecting AZ31 magnesium alloy tubes (AMATs) with radioactive ^125^I seeds. A secondary aim was to evaluate whether this innovative configuration could provide structural support for the ^125^I seeds, simplify the implantation process, and reduce both implantation time and radiation exposure for interventional radiologists.

## 2 Results and Discussion

### 2.1 Characterization of AMASS

First, we measured the mechanical parameters of AMATs, including the axial maximum force, bending strength, elastic modulus, and connection strength with radioactive ^125^I seeds (**Table** **1**). The axial maximum force of AMATs was 88.83 ± 6.24 N, indicating sufficient compressive strength to prevent deformation during ^125^I seed strand implantation. Additionally, the AMATs’ bending strength was 204.67 ± 13.61 MPa, and its elastic modulus was 12 280.00 ± 3611.15 MPa, demonstrating substantial rigidity and resistance to bending under external forces. Notably, the connection strength between AMATs and ^125^I seeds was 22.85 ± 3.15 N. This ensures that the insertion force does not displace the seeds, maintaining a fixed spacing and facilitating uniform dose distribution.

**Table 1:** Mechanical parameters of AZ31 magnesium alloy seed strands.

Sample	Axial maximum force [N]	Bending strength [MPa]	Elastic Modulus [MPa]	Connection strength with ^125^I seeds [N]
1	86.84	200	13 260	26.48
2	95.82	220	8280	20.89
3	83.82	194	15 300	21.18
Mean ± SD	88.83±6.24	204.67 ± 13.61	12 280.00 ± 3611.15	22.85 ± 3.15

**Figure 2:**
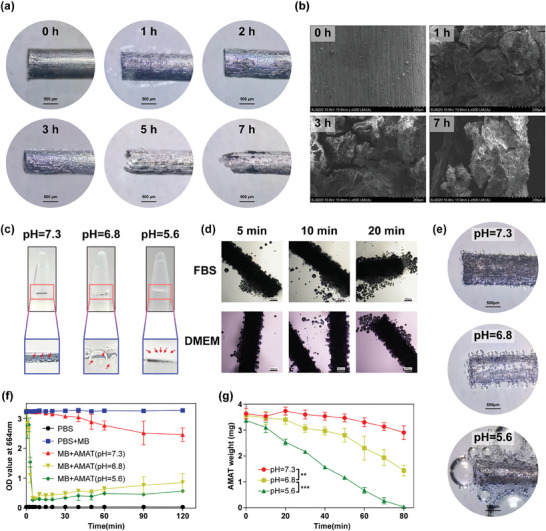
Corrosion and hydrogen evolution characteristics of AZ31 magnesium alloy tubes (AMAT). Surface characteristics of AMAT at various time points post‐corrosion under the a) stereomicroscope and b) scanning electron microscopic. c) Visual observation of hydrogen evolution capability under different pH conditions. d) Microscopic observation of hydrogen evolution ability at different time points in fetal bovine serum (FBS) and DMEM culture media. e) Stereomicroscopic observation of hydrogen evolution capability under different pH conditions. f) Relative hydrogen evolution rates under pH conditions of 7.3, 6.8, and 5.6 determined by the methylene blue (MB) method. g) Weight loss of AMATs under different pH values. Data are shown as the mean ± standard deviation. Statistical differences are represented as *p < 0.05, **p < 0.01, ***p < 0.001; ns, *p* > 0.05, indicates no significant difference.

To observe the surface characteristics and corrosion morphology of AMATs, we examined AMATs soaked in phosphate‐buffered saline (PBS) (pH 6.8) for 0, 1, 3, or 7 h using optical microscopy and scanning electron microscopy (SEM) (**Figure** **2**a,b). The results indicated that corrosion on the AMAT surface progressively worsened over time. At ≈1 h, small pits appeared on the surface, which expanded into large flaky depressions after 2 h. After 3 h, the AMAT surface did not have smooth areas left, and significant damage was visible at the tube ends after 5 h. By 7 h, the damage had spread along the lengths of the tubes. These results indicate that AMATs gradually degraded over time in mildly acidic environments.

According to the literature, the pH of the normal tissue stroma ranges from 7.3 to 7.4, whereas the pH inside human tumors generally falls between 6.4 and 7.0, with the lowest observed value being 5.6.^[^ ^24^ ^]^ To evaluate the hydrogen production capacity of the AMATs, we immersed them in PBS solutions with pH values of 7.3, 6.8, and 5.6. After 5 min of immersion, a visual inspection revealed that the surface of the magnesium tube in the pH 7.3 group produced only a few bubbles with minimal hydrogen diffusion into the solution. In contrast, the pH 6.8 group showed larger bubbles and moderate hydrogen diffusion, while the pH 5.6 group exhibited rapid hydrogen production, with numerous small bubbles quickly dispersing into the solution. These results indicate a faster hydrogen production rate at lower pH values (Figure 2c). Further microscopic observation of AMATs placed in normal serum and cell culture medium for 5, 10, and 20 min demonstrated an increase in hydrogen production over time, which was particularly prominent at the opening of the tube (Figure 2d). Stereomicroscopy analysis confirmed that the hydrogen production capacity of AMATs increased with decreasing pH (Figure 2e).

Next, to quantify hydrogen production in AMATs, we measured the optical density (OD) of methylene blue (MB) solutions at 664 nm. The results showed that the OD for the pure PBS group was close to zero, whereas the PBS + MB group maintained an OD value of ≈3.2, confirming the stability of the MB solution. The OD values began to decrease upon introducing AMATs into the reaction system, indicating hydrogen production. The decrease in OD was slow in the pH 7.3 group. In contrast, in the pH 6.8 and pH 5.6 groups, the OD values dropped rapidly within the first 5 min and then stabilized, with the pH 5.6 group consistently exhibiting lower OD values due to its stronger acidity than the pH 6.8 group (Figure 2f). Finally, we indirectly quantified the volume of hydrogen produced by measuring the weight loss of AMATs under different pH conditions (Figure 2g; Figure S1, Supporting Information). AMATs generate hydrogen upon exposure to water. The hydrogen production rate increases as the pH decreases, indicating more pronounced corrosion and accelerated hydrogen production at lower pH levels.

Hydrogen is widely used in clinical treatments and basic research. In this study, our qualitative and quantitative analyses confirmed the hydrogen production capacity of the AMATs. Various methods are currently employed for hydrogen delivery in disease treatment. The direct inhalation of hydrogen gas,^[^ ^25^ ^]^ which is limited by hydrogen's poor solubility in water and lack of appropriate blood carriers, makes it difficult to transport hydrogen to distant tumors. Drinking hydrogen‐rich water,^[^ ^26^ ^]^ which, while safe, is limited by the low solubility of hydrogen in water and its poor absorption in the digestive tract. Next is the injection of hydrogen‐saturated saline;^[^ ^27^ ^]^ however, it requires large volumes to achieve therapeutic hydrogen concentrations due to its low solubility, limiting its use to small animals. Hydrogen‐loaded microbubbles can be controlled by ultrasound,^[^ ^28^ ^]^ which increases local hydrogen concentrations but suffers from low hydrogen content and poor stability, making it unsuitable for long‐term tumor treatment. The intravenous injection of hydrogen‐releasing nanomaterials is another method.^[^ ^29^ ^]^ It involves encapsulating hydrogen‐producing substances in nanomaterials for controlled release, although targeting tumors using the technique remains challenging. The arterial injection of hydrogen‐releasing nanoparticles mixed with iodized oil for liver tumor embolization,^[^ ^30^ ^]^ which increases local hydrogen content and minimizes damage to normal tissue, has also been demonstrated but is only suitable for liver tumors. Finally, there have been reports on the microneedle delivery of hydrogen,^[^ ^31^ ^]^ which releases hydrogen locally for skin cancer or melanoma treatment but is not suitable for deep‐seated tumors. Our study demonstrated that AMATs offer several advantages over these methods because they can be implanted alongside ^125^I seeds via interventional procedures to release hydrogen locally in tumors; the degradation period of AMATs matches the effective duration of ^125^I seeds, lasting ≈3–4 weeks, which is ideal for tumor treatment; and the biodegradation of AMATs produces Mg^2+^, a common ion in the human body that can be safely metabolized without adverse effects.

Because Mg reacts with water to produce both H_2_ gas and OH^−^ ions, which increase the pH of the solution, we investigated the effect of AMAT degradation on the pH of the solution at pH 6.8. After AMAT immersion, the pH of the solution began to increase. The initial fluctuations were likely due to measurement errors from the short intervals between readings. Once measurements were taken every 10 min, the pH steadily increased until the AMAT was fully corroded after ≈3 h, stabilizing at ≈8.3. The overall pH change was ≈1.51, indicating that the hydroxide ions from the AMAT degradation increased the pH of the solution (Figure S2, Supporting Information). Therefore, we hypothesized that placing AMATs in the slightly acidic microenvironment of a tumor could neutralize its acidity, potentially leading to therapeutic effects.

### 2.2 Role of Hydrogen Production by AMATs in Enhancing Tumor Cell Radiosensitivity

**Figure 3:**
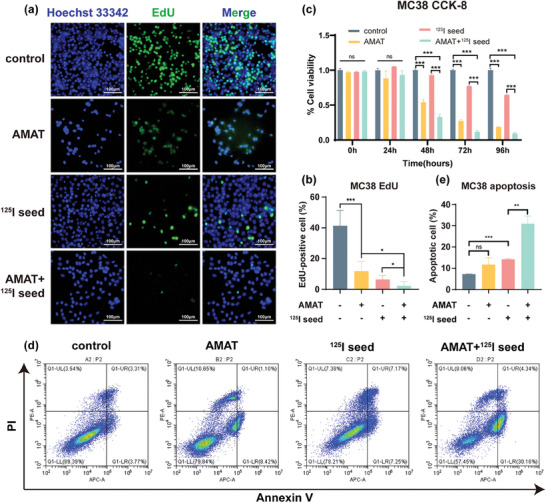
The role of AZ31 magnesium alloy tubes (AMAT) in modulating the radiosensitivity of tumor cells to ^125^I seeds. a,b) Percentage of EdU‐positive MC38 cells after treatment with AMAT and ^125^I seeds. c) Cell viability of MC38 cells after treatment with AMAT and ^125^I seeds. d,e) Apoptotic rate of MC38 cells after treatment with AMAT and ^125^I seeds. Data are shown as the mean ± standard deviation. Statistical differences are represented as *p < 0.05, **p < 0.01, ***p < 0.001; ns, *p* > 0.05, indicates no significant difference.

Because of the reaction between Mg and water, hydrogen gas and a certain amount of Mg^2+^ are produced. To eliminate the influence of Mg^2+^ on tumor cell proliferation, we first measured the concentration of Mg^2+^ in the supernatant of cell cultures containing AMATs, which was found to be 5.08 ± 0.02 mm (Table S1, Supporting Information). Consequently, we prepared a cell culture medium containing 5.0 mm Mg^2+^ by diluting a 2 mol L^−1^ MgCl_2_ solution in a conventional cell culture medium. CCK‐8 assays revealed that 5.0 mm Mg^2+^ did not affect the proliferation of the three tumor cell lines compared with the control group (Figure S3, Supporting Information). Next, using an in vitro hydrogen brachytherapy device, we examined the effects of AMATs and radioactive ^125^I seeds, both alone and in combination, on tumor cells. First, EdU incorporation assays were performed to assess the proliferation of tumor cells. Cell nuclei were stained with Hoechst 33 342 and EdU conjugated to an Apollo 488 dye. As shown in **Figure** **3**a,b, the proportion of EdU‐positive MC38 cells was significantly decreased in the AMAT and ^125^I seed groups compared to that in the control group. Moreover, the combined treatment group had an even lower proportion of EdU‐positive cells than the ^125^I seed group alone, indicating that AMATs enhanced the inhibitory effect of ^125^I seeds on tumor cell proliferation. Similar results were observed for the other two tumor cell lines (Figure S4, Supporting Information). Notably, in VX2 tumor cells, there was no statistically significant difference between the ^125^I seed and combined treatment groups. This discrepancy may be due to the apoptosis induced in the combined treatment group, causing cells to detach and float, leading to a reduced number of adherent cells on the culture plate and an artificially high proportion of EdU‐positive cells. CCK‐8 assays were also conducted to observe the effects of the hydrogen gas produced by AMATs on tumor cell proliferation. Compared with the control group, cell viability was significantly decreased in the AMAT and ^125^I seed groups, with the combined treatment being more effective than either treatment alone (Figure 3c; Figure S5, Supporting Information). Specifically, the onset of this effect varied among the three tumor cell lines: 72 h for HepG2, 48 h for MC38, and 24 h for VX2 cells, potentially due to the inherent differences in the radiosensitivity of these cell lines. Overall, these results indicated a synergistic effect of hydrogen produced by AMATs and radiation from ^125^I seeds on inhibiting tumor cell proliferation.

We further investigated the effects of AMATs and ^125^I seeds on tumor cell apoptosis. First, we investigated the effect of Mg^2+^ on the apoptosis rate of three tumor cell lines. The results indicated that 5 mm Mg^2+^ did not significantly affect the apoptosis rates of the cell lines investigated (Figure S6, Supporting Information). Subsequently, we treated the tumor cells with AMATs in combination with radioactive ^125^I seeds and assessed cell apoptosis using flow cytometry. The results demonstrated that both AMATs and ^125^I seeds alone promoted apoptosis in all three tumor cell lines, with a significant increase in apoptosis rates observed in the combined treatment group (Figure 3d,e; Figure S7, Supporting Information). These findings suggest that the hydrogen produced by AMATs can enhance the pro‐apoptotic effects of ^125^I seed irradiation in the three tumor cell lines we investigated.

Additionally, to investigate the role of AMATs in external beam radiation therapy, we also conducted flow cytometry experiments (Figure S8, Supporting Information) and γH2A.X immunofluorescence assays (Figure S9, Supporting Information). The results indicated that the hydrogen gas produced by AMAT degradation enhances the pro‐apoptotic effect of external beam radiation on tumor cells. Moreover, AMATs increased the DNA damage inflicted on tumor cells by external beam radiation. We also validated the radiosensitizing effect of AMAT in CT26 colorectal cancer cells, a type of microsatellite‐stable tumor cell (Figure S10, Supporting Information). The results show that AMATs enhanced the pro‐apoptotic effect of external beam radiation on CT26 cells.

### 2.3 Mechanism of AMATs in Enhancing Radiosensitivity in Solid Tumors

**Figure 4:**
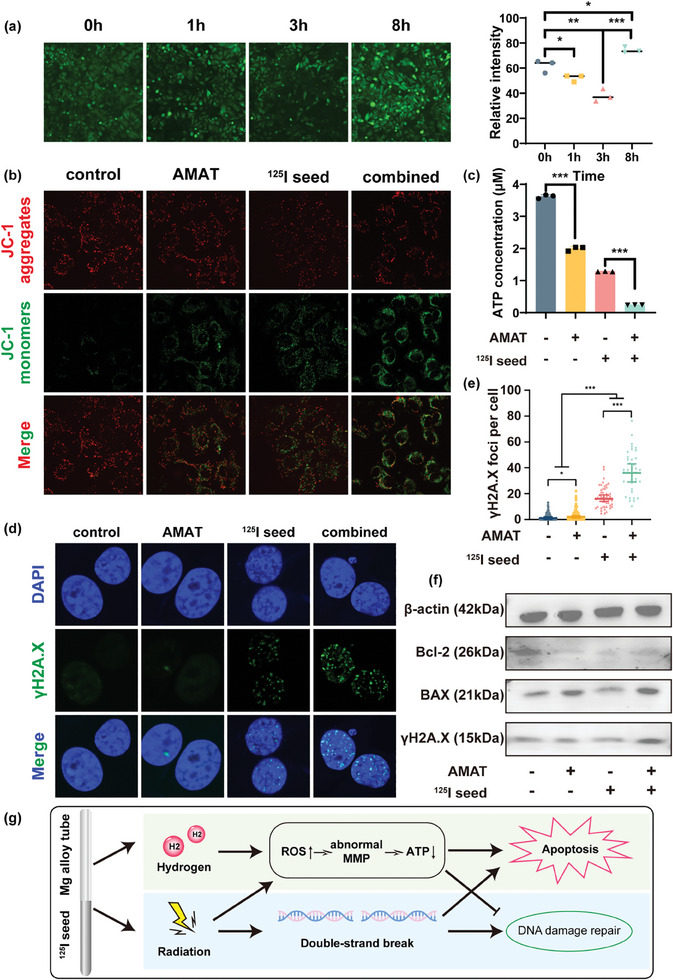
Mechanism of AZ31 magnesium alloy tubes (AMAT) in enhancing radiosensitivity in solid tumor cells. a) ROS levels in VX2 cells at different time points following AMATs treatment. b) Mitochondrial membrane potential of VX2 cells following treatment with AMATs and ^125^I seeds. c) Intracellular ATP levels of MC38 cells following treatment with AMATs and ^125^I seeds. d,e) γH2A.X levels of MC38 cells following treatment with AMATs and ^125^I seeds. f) BAX and Bcl‐2 levels of VX2 cells following treatment with AMATs and ^125^I seeds. g) In vitro mechanism diagram of the hydrogen brathytherapy. Data are shown as the mean ± standard deviation. Statistical differences are represented as *p < 0.05, **p < 0.01, ***p < 0.001; ns, *p* > 0.05, indicates no significant difference.

Hydrogen gas is involved in cancer treatment via multiple pathways, with two primary mechanisms being the most studied and widely accepted. First, hydrogen gas modulates ROS levels in tumor cells, disrupting the redox balance and promoting apoptosis.^[^ ^19^, ^32^ ^]^ Second, as a reducing agent, hydrogen gas targets the mitochondrial oxidative respiratory chain, causing abnormalities in the mitochondrial membrane potential (MMP) and mitochondrial dysfunction, leading to decreased intracellular adenosine triphosphate (ATP) levels and inhibition of tumor growth.^[^ ^33^ ^]^ Thus, we hypothesized that the hydrogen generated from AMATs might regulate ROS levels within tumor cells, causing redox stress and subsequently disrupting mitochondrial function. First, we assessed the intracellular ROS levels at different time points after AMAT treatment. The results indicated that although the ROS levels gradually decreased within the first 3 h, a resurgence in ROS levels was observed at the 8 h mark, surpassing the pre‐treatment levels (**Figure** **4**a; Figure S11, Supporting Information). This may be attributed to the reductive hydrogen neutralizing intracellular ROS, leading to redox stress in the tumor cells. Furthermore, we measured mitochondrial membrane potential. The control cells predominantly displayed red JC‐1 aggregates, indicating healthy mitochondria. In contrast, cells treated with AMATs or radioactive ^125^I seeds exhibited a balanced presence of red aggregates and green JC‐1 monomers, suggesting partial mitochondrial dysfunction. Notably, cells in the combination treatment group showed a significant increase in the number of green monomers over red aggregates, indicating severe MMP disruption and extensive mitochondrial damage compared to either treatment alone (Figure 4b). This suggests that hydrogen from AMATs enhances the mitochondrial dysfunction induced by ^125^I seeds. Given that mitochondria are a primary site of ATP production within cells, we assessed intracellular ATP levels following treatment with AMAT and ^125^I seeds. The results demonstrated a significant reduction in intracellular ATP levels following treatment with either AMAT or ^125^I seeds, with an even more pronounced decrease observed in the combination treatment group (Figure 4c; Figure S12, Supporting Information).

^125^I seeds are known to cause DNA double‐strand breaks (DSBs), leading to DNA damage and tumor cell apoptosis.^[^ ^34^ ^]^ Phosphorylation of histone H2A.X to form γH2A.X is a sensitive marker of DSBs.^[^ ^35^ ^]^ Therefore, we performed γH2A.X immunofluorescence staining to assess the DSBs induced by ^125^I seeds in the presence of hydrogen from the AMATs. First, immunofluorescence assay demonstrated that 5 mm Mg^2+^ has no effect on the DNA damage of HepG2 and VX2 cells (Figure S13, Supporting Information). Additionally, hydrogen alone did not significantly increase the number of DSBs. However, in ^125^I‐irradiated cells, the number of DSBs increased significantly, and the addition of hydrogen further elevated γH2A.X foci, indicating enhanced DSBs (Figure 4d,e; Figure S14, Supporting Information). This demonstrates that hydrogen from magnesium alloy tubes exacerbates ^125^I‐induced DNA damage.

Next, we extracted proteins from the treated tumor cells and performed western blotting to assess the expression of DNA damage‐ and apoptosis‐related molecular markers. Compared to the control group, the expression of the anti‐apoptotic protein B‐cell lymphoma 2 (Bcl‐2) was significantly reduced, whereas that of the pro‐apoptotic protein Bcl‐2‐associated X protein (BAX) was significantly increased. This suggests that the hydrogen generated from AMATs, when combined with ^125^I seeds, induces a pro‐apoptotic shift in the tumor cells (Figure 4f). In addition, there were no significant differences in the expression levels of caspase‐3 among the four groups. However, the expression of cleaved caspase‐3, the active form of caspase‐3, significantly increased in the combined treatment group of AMATs and ^125^I seeds, indicating increased apoptosis (Figure S15, Supporting Information). Furthermore, we also confirmed the expression of the DSB‐sensitive marker γH2A.X in tumor cells. Consistent with the immunofluorescence results, the expression of γH2A.X in the combined treatment group was significantly higher than that in the control and ^125^I seed‐only groups (Figure 4f; Figure S15, Supporting Information).

In conclusion, hydrogen from AMATs potentiates the therapeutic effects of ^125^I seeds through two mechanisms: 1) disruption of the MMP and induction of apoptosis, and 2) enhancement of radiation‐induced DNA damage by inhibiting the DDR process, ultimately leading to tumor cell apoptosis (Figure 4g).

### 2.4 Therapeutic Effects of AMATs on Subcutaneous Xenograft Tumors in Mice

**Figure 5:**
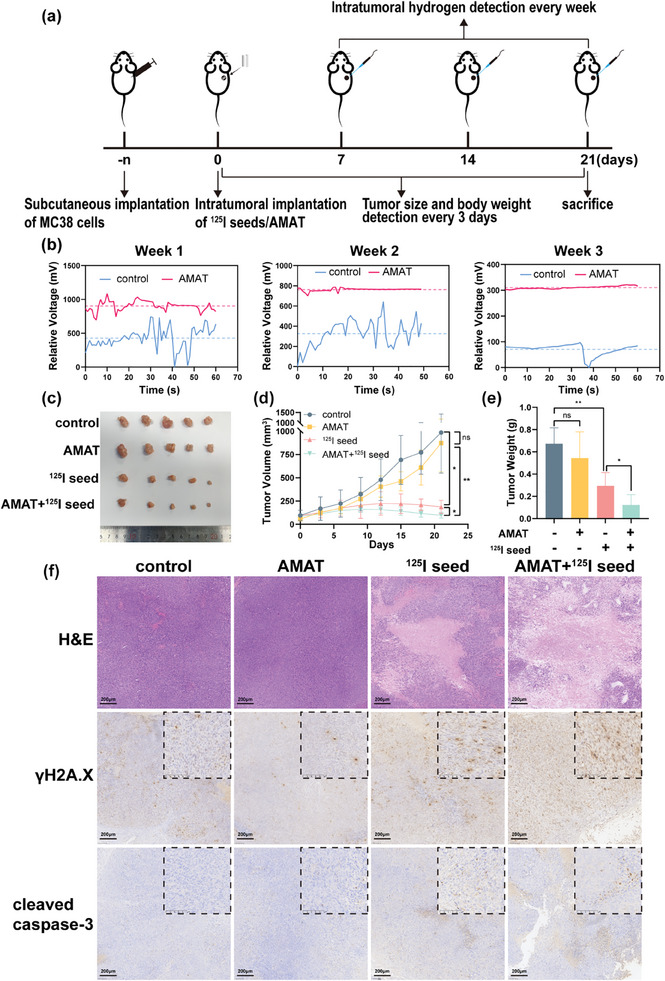
Therapeutic effects of AZ31 magnesium alloy tubes (AMATs) on subcutaneous xenograft tumors in mice. a) Experimental procedure diagram for subcutaneous tumor transplantation in mice. b) Comparison of hydrogen content in subcutaneous tumor implants of mice at 1, 2, and 3 weeks after implantation of AMATs. c,d) Volume of subcutaneous tumors in mice. e) Tumor weight statistical chart. f) H&E staining and IHC of subcutaneous transplanted tumors in mice. Data are shown as the mean ± standard deviation. Statistical differences are represented as *p < 0.05, **p < 0.01, ***p < 0.001; ns, *p* > 0.05, indicates no significant difference.

To demonstrate the ability of AMATs to generate hydrogen gas within the tumor microenvironment, we utilized H_2_ microelectrodes to measure hydrogen levels in tumors from mice implanted with AMATs compared with control mice. Measurements were taken at 1, 2, and 3 weeks post‐implantation (**Figure** **5**a; Figure S16, Supporting Information). The results indicated that the hydrogen levels in the tumors of the AMAT group were consistently higher than those in the control group at all time points. The relative voltage differences between the AMAT and control groups were ≈475, 436, and 240 mV in the first, second, and third weeks, respectively (Figure 5b). The noticeable decrease in the relative voltage difference in the third week was likely due to corrosion and a reduction in the size of the AMATs, resulting in a decreased surface area in contact with water and reduced hydrogen production. These findings confirmed that the implanted AMATs effectively generated hydrogen gas within the tumor microenvironment, particularly during the initial weeks post‐implantation. Furthermore, these results suggest that hydrogen generation was essential for the proposed synergistic therapeutic effects of ^125^I seeds in inhibiting tumor growth and promoting apoptosis.

We observed that the degradation time of AMATs in vivo differed from that in the cell culture medium, with in vivo degradation being slower, taking ≈3–4 weeks for complete degradation. This discrepancy may be attributed to the robust buffering systems and complex tumor microenvironment in animals. Factors, such as the presence of various cells, concentrations of organic substances and cytokines, and blood flow rates within the tumor, can influence the degradation of magnesium in vivo. Furthermore, compared to other magnesium alloys, such as ZK40 and high‐purity magnesium single‐crystal Mg8H, the degradation rate of the AZ31 magnesium alloy is considerably slower.^[^ ^36^ ^]^ The corrosion rate of the AZ31 magnesium alloy tubes aligned with our expectations, providing sufficient support for the seeds while allowing gradual biodegradation. Given the low molecular weight and high permeability of hydrogen gas, some studies have measured hydrogen levels on the skin surface of nude mice and found similar results to those obtained within the gas cavity.^[^ ^37^ ^]^ In our study, considering the black fur of the *C57BL/6J* mouse strain, the slow degradation rate of AMATs, and the implantation site within the tumor rather than under the skin (subcutaneous), we chose to measure hydrogen gas levels by inserting the electrode directly into the tumor tissue after anesthetizing the mice.

To investigate the safety of combining hydrogen production from AMATs with ^125^I seeds in treating subcutaneous tumors in mice, we monitored the body weight of the mice every 3 d post‐implantation. The results showed no significant weight loss in mice from the AMAT, ^125^I seed, or combined treatment groups compared to the control group (Figure S17, Supporting Information). To assess the efficacy of this combined treatment, we measured the tumor size with calipers every 3 d. The results indicate that the tumor volumes in both groups implanted with ^125^I seeds were smaller than those without ^125^I seeds. Furthermore, the combined AMAT and ^125^I seed treatment group exhibited more significant tumor inhibition than the ^125^I seed‐only group, demonstrating the synergistic effect of hydrogen production on ^125^I seed radiotherapy (Figure 5c,d). Additionally, the postmortem tumor weights corroborated these findings (Figure 5e). Notably, there was no statistically significant difference in tumor volume and tumor weight between the control and AMAT‐only groups. This lack of difference might be due to the stronger stress response of tumor cells in vivo than in vitro. Thus, without ^125^I radiation, tumor cells may resist the reducing effects of hydrogen produced by AMATs. Additionally, due to ethical regulations concerning tumor size in mice, the tumors in the control group approached the size limit around the third week. Consequently, we collected tumor samples on day 21. Although the differences were not dramatic, they did demonstrate that AMAT has a radiosensitizing effect. The half‐life of the ^125^I radionuclide is 59.6 days, so if conditions allow for continued observation, the differences between the two groups may become more pronounced over time. However, under ^125^I radiation, DNA damage in tumor cells allows the reduction of hydrogen to enhance radiotherapy, thereby acting as a radiosensitizer.

Previous studies have found that implanting pure magnesium wires into mouse tumors significantly inhibits tumor growth.^[^ ^38^ ^]^ However, our animal experiments showed no significant difference in tumor growth between the group implanted with AMATs alone and the control group. This discrepancy can be attributed to several factors. First, the magnesium materials used differed across studies; we used the AZ31 magnesium alloy in this study, which degrades slower than the high‐purity magnesium used in other studies. Second, we used a different cell line, the slow‐growing mouse MC38 colon cancer cell line, instead of a human tumor cell line. Lastly, the in vivo tumor microenvironment differed from in vitro conditions. Therefore, the hydrogen gas from AMATs alone may not inhibit tumor growth in vivo. However, when combined with ^125^I seed radiation, which damages the tumor cell DNA, the hydrogen gas enhanced tumor radiosensitization.

Additionally, we performed hematoxylin and eosin (H&E) staining and immunohistochemistry on the subcutaneous tumors of mice. As shown in Figure 5f, the group treated with ^125^I seeds and AMATs exhibited extensive necrosis. Although the ^125^I seed group showed higher expressions of γH2A.X and cleaved caspase‐3 than the control group, the combined treatment group had even higher expressions of these markers. These results confirm that hydrogen produced by AMATs can enhance ^125^I seed‐induced DNA damage and apoptosis in vivo.

### 2.5 Therapeutic Effects of AMASSs on Liver Tumors in New Zealand Rabbits

**Figure 6:**
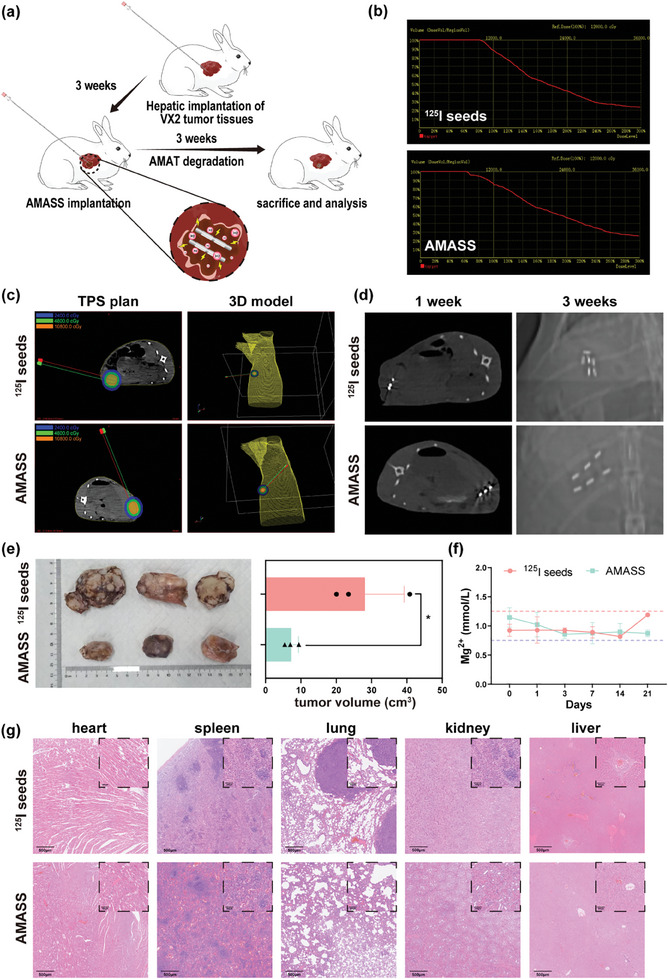
Therapeutic effects of AZ31 magnesium alloy ^125^I seed strands (AMASSs) on liver tumors in New Zealand rabbits. a) Experimental model diagram of liver tumor in New Zealand rabbits. b) Preoperative dose volume histogram (DVH) from treatment planning system (TPS). c) Isodose curve and 3D model diagram of preoperative TPS plan. d) The distribution of radioactive ^125^I seeds three weeks after implantation of AMASS or ^125^I seeds. e) Tumor volume three weeks after implantation of ^125^I seeds or AMASS. f) Changes in blood magnesium ion levels after implantation of ^125^I seeds or AMASS. g) H&E staining of major organs three weeks after implantation of ^125^I seeds or AMASS. Data are shown as the mean ± standard deviation. Statistical differences are represented as *p < 0.05, **p < 0.01, ***p < 0.001; ns, *p* > 0.05, indicates no significant difference.

Owing to the size limitations of subcutaneous xenografts in mice, AMATs and ^125^I seeds could only be placed side by side rather than forming AMASS. Therefore, we established a rabbit liver tumor model to explore the dosage and therapeutic effects of AMASS (**Figure** **6**a). To ensure the establishment of liver tumor models and complete subsequent treatment planning system (TPS) planning, we performed a liver computed tomography (CT) scan on New Zealand rabbits 1 d before the implantation of the AMASSs. The CT scan showed a round, low‐density area on the liver adjacent to the abdominal wall, indicating the presence of a tumor (Figure S18, Supporting Information). On the same day, we completed the preoperative TPS plan. Our calculations showed the need for placing two rows of seed strands, each containing three 0.8 mCi ^125^I seeds and/or two AMATs to meet the prescribed dose. The dose and volume histogram (DVH) revealed that 90% of the tumor region received a dose (D90) of 120 Gy (Figure 6b). The isodose curves and 3D model indicated that the radiation field of the radioactive ^125^I seeds closely matched that of the gross tumor volume (GTV) (Figure 6c). Notably, the 4800 cGy isodose curve extended to the skin; however, we believe this was due to the mobility of the liver during normal animal activity, which differed from its position during the CT scan, along with the continuous motion of the ^125^I seeds. Consequently, it did not result in skin tissue damage. Three weeks after implantation of the seeds or AMASSs, no symptoms of radiation dermatitis, such as skin redness or ulceration, were observed in the rabbits.

An inconsistent dose distribution is one of the reasons for the suboptimal efficacy of ^125^I seed therapy. This inconsistency arises as the tumor volume changes, causing the ^125^I seeds to shift in position. To monitor the relative position of the ^125^I seeds, CT scans were performed 1 and 3 weeks after the implantation of the AMASSs. The CT images at 1 week showed a uniform distribution of ^125^I seeds in the AMASS group, whereas the individual ^125^I seed group exhibited an uneven distribution with variable spacing (Figure 6d). This uneven distribution could result from uneven spacing during the manual implantation process or the post‐implantation migration of the seeds. Regardless of the cause, AMASSs inhibited seed migration. Furthermore, CT images at 3 weeks post‐implantation clearly showed significant migration in the individual ^125^I seed group, worsening from the first week. In contrast, the AMASS group displayed minimal migration, with only one seed slightly displaced (lower right), which was attributed to the corrosion and breakage of the AMATs after 3 weeks (Figure 6d). However, due to the metallic artifacts from the 125I seeds and the X‐ray visibility of the magnesium alloy, we were unable to obtain clear images of AMAT on the CT scans. These results indicated that AMATs effectively prevented the migration of ^125^I seeds and eventually degraded them to allow better conformity between the seeds and the tumor.

To evaluate the efficacy of AMASSs in treating liver tumors in New Zealand rabbits, a CT scan was performed at 3 weeks post‐implantation. Following the scan, the rabbits were euthanized, and liver tumor tissues were extracted. As shown in Figure 6e, the tumor volume in the AMASS group was significantly lower than that in the ^125^I seed group. These findings suggest that the hydrogen produced by AMATs and the uniform distribution of the seeds may enhance the therapeutic efficacy of ^125^I seeds, effectively reducing the tumor burden in the liver.

To assess the safety of AMASSs for treating liver tumors in rabbits, blood samples were collected at 1, 3, 7, 14, and 21 d post‐implantation. The blood tests performed included a complete blood count, liver and kidney function tests, and serum magnesium ion concentration measurements. As shown in Figure S19 (Supporting Information), the red blood cell, white blood cell, platelet, and neutrophil counts remained stable and within normal ranges after implantation. Liver function tests indicated no significant increases in alanine aminotransferase (ALT) and aspartate aminotransferase (AST), levels, which remained within normal limits. Kidney function tests also showed no significant elevation in creatinine (CREA) and urea nitrogen (UREA) levels, which remained within normal ranges. Additionally, serum magnesium ion levels did not increase (Figure 6f), suggesting that the magnesium ions released from the degrading alloy were likely excreted via the urine, consistent with previous studies.^[^ ^37^ ^]^ These results demonstrate that AMASS implantation did not adversely affect hematopoietic function, liver function, kidney function, or serum magnesium ion levels in New Zealand rabbits.

To further investigate the safety profile of AMASSs in the treatment of liver tumors, key organs, including the heart, spleen, lungs, kidneys, and liver, were harvested from euthanized New Zealand rabbits 3 weeks post‐implantation and subjected to H&E staining. The results indicated no significant toxic effects of AMASS on these major organs. Similarly, the implantation of ^125^I seeds alone did not result in noticeable organ toxicity. However, the lung tissue sections from rabbits treated with ^125^I seeds alone showed evidence of pulmonary metastasis (Figure 6g). These findings suggest that while both treatments exhibited good safety profiles regarding major organ toxicity, AMASSs may offer an additional benefit in reducing metastatic spread, thus demonstrating the overall enhanced safety and efficacy in the treatment of liver tumors in New Zealand rabbits.

Compared to other seed/space linking strategies, AMASS offers several advantages. First, AMASS degrades within the tumor to produce hydrogen gas, thereby enhancing radiosensitivity. Additionally, the connectors in other seed chains are often made of poly(lactic‐co‐glycolic acid) (PLGA), which has a prolonged degradation time and may not adapt well to tumor shrinkage. If the tumor reduces in size quickly, the ^125^I seeds on the tumor's outer edge may shift into surrounding healthy tissue due to the support provided by PLGA, increasing treatment risk. In contrast, AMAT degrades in ≈3–4 weeks, aligning more closely with the observed tumor regression rate in clinical settings.

However, this study has several limitations. First, post‐operative TPS validation was not performed after implanting ^125^I seeds or AMASS in New Zealand rabbit liver tumors. The larger size of rabbits precluded micro‐CT scanning, so we used a 16‐slice CT scanner for post‐operative observation. However, significant metal artifacts from ^125^I seeds, even in the bone window setting, obscured the tumor's position and size, preventing GTV delineation. Thus, post‐operative TPS validation was compromised by CT image clarity and metal artifacts. Future studies should consider larger animal models for AMASS implantation and validation. Second, we followed up for only three weeks post‐implantation until the AMATs degraded, but the long‐term safety beyond this period remains unknown due to the continued radiation from the seeds. Additionally, the tumor cell lines used were of animal origin, which may not fully represent human tumor pathology and growth patterns. Thirdly, the assembly of AMASS was manually performed before implantation and has not yet been produced on a large scale. Despite lead gloves and a lead‐shielded table, radiation exposure to the radiologists occurred. Future research will focus on mechanical assembly designs for the AMASS to mitigate this issue. Lastly, safety studies for this product have not been conducted. In future research, safety assessments will be carried out as part of preclinical studies prior to application in humans.

## 3 Conclusion

In summary, we found that AMATs had the appropriate rigidity and good connectivity with ^125^I seeds. More importantly, AMATs could degrade in solution to produce hydrogen gas, and the hydrogen production rate was pH‐dependent. We further explored the role and potential molecular anticancer mechanisms of hydrogen gas produced by AMAT degradation in enhancing the treatment of solid tumors with radioactive ^125^I seeds. Functionally, the hydrogen produced by the degradation of AMAT could synergize with ^125^I seeds, inhibiting tumor cell proliferation and promoting apoptosis. Mechanistically, hydrogen disrupted ROS homeostasis and MMPs in tumor cells, impaired mitochondrial function, reduced ATP levels, and obstructed the repair of DSBs induced by ^125^I radiation, thereby enhancing the radiosensitization effect of ^125^I seed therapy in solid tumors. This study improved the efficacy of brachytherapy for solid tumors through two approaches: 1) the hydrogen produced by the AMAT synergized with the radiation from the ^125^I seeds, and 2) the AMASS enhanced the spatial arrangement of the seeds, resulting in a more uniform dose distribution. These results highlight the potential of AMATs for improving the efficacy of ^125^I seed brachytherapy and expanding its clinical application range. Future research will focus on using RNA sequencing to explore the specific molecular pathways by which hydrogen released from AMASS enhances radiosensitivity.

## 4 Experimental Section

### Preparation of AMASS

In the previous study, a controllable, degradable magnesium alloy seed strand was developed composed of hollow AZ31 magnesium alloy connecting tubes and 6711‐type radioactive ^125^I seeds connected alternately.^[^ ^39^ ^]^ The AZ31 magnesium alloy connecting tubes were manufactured by Sanming Medical Equipment Co., Ltd. (Yangzhou, China), with the following specifications: inner diameter, 0.8 mm; wall thickness, 0.1 mm; and length, 6.0 mm. The AZ31 alloy consisted of Mg (95.8% w/w), Al (3.0% w/w), Zn (0.8% w/w), and Mn (0.4% w/w). The magnesium alloy connecting tubes were sterilized with ethylene oxide, vacuum‐packed, and irradiated with ultraviolet light overnight before use. The 6711‐type radioactive ^125^I seeds were purchased from Zhibo Bio‐medical Technology Co., Ltd. (Beijing, China). They had a diameter of 0.8 mm, a length of 4.5 mm, and an average activity of 29.6 × 10^6^ Bq (0.8 mCi) per seed. All seeds were subjected to quality inspection and sterilization before shipment.

### Mechanical Characterization of AMASS

The mechanical properties of the magnesium alloy seed strands were measured by CVC Testing Technology Co., Ltd. (Guangzhou, China). The key parameters evaluated included the axial maximum deformation force, bending strength, elastic modulus, and connection strength between the magnesium alloy tube and the ^125^I seed. All measurements were conducted in accordance with the GB/T 228.1‐2021 Standard for Metallic Materials Tensile Testing.

### Observation of the Surface Corrosion of AMATs

The AMATs were immersed in PBS (pH = 6.8; Procell Technology Co., Ltd., Wuhan, China) for 0, 1, 2, 3, 5, or 7 h. The surface structures of the corroded magnesium alloy tubes were observed using SEM (FEI, Waltham, MA, USA) and stereomicroscopy (Olympus, Tokyo, Japan).

### Design of an In Vitro Hydrogen Radiotherapy Device

To construct this device, a 0.8 µm Transwell insert (Corning, New York, USA) was placed on top of each well of 6‐well or 24‐well cell culture plates. Tumor cells were cultured in the upper compartment of the insert, whereas AZ31 magnesium alloy tubes and radioactive ^125^I seeds were placed in the lower compartment. Both the upper and lower compartments were filled with an appropriate volume of cell culture medium. The hydrogen gas produced by the degradation of the magnesium alloy tubes could rise and come into contact with the tumor cells, and the radiation from the ^125^I seeds could irradiate the tumor cells, thereby achieving a dual treatment effect. A schematic diagram of the device is shown in Figure S20 (Supporting Information).

### Measurement of Hydrogen Production by AMATs

The hydrogen production capability of the AMATs was measured in vitro using the MB reduction method. MB solution was purchased from Sangon Biotech Co., Ltd. (Shanghai, China). In a 96‐well plate, PBS solutions (with different pH) and MB were added to each well. Subsequently, the Mg alloy tubes were immersed in the wells, and the absorbance of the solution in each well at 664 nm was measured at different time points using a VersaMax full‐wavelength microplate reader (Molecular Devices, San Jose, CA, USA) (Figure S21, Supporting Information).

The hydrogen content in the animal models was measured using a hydrogen microelectrode (Unisense, Aarhus, Denmark). Mice in the control and magnesium alloy tube groups were anesthetized. A polarized hydrogen microelectrode was inserted into the subcutaneously transplanted tumor in the mice, and voltage changes were observed and recorded.

### Weight Loss Measurement of AMATs

Briefly, the AMATs were immersed in PBS solutions with different pH values. Every 10 min, the tubes were removed, blotted dry with lint‐free paper, and weighed. After weighing, the tubes were reimmersed in the corresponding solution. This process was repeated until AMATs were fully degraded. The weight loss of the magnesium alloy tubes was converted into the volume of hydrogen gas produced using Equations (1) and (2), as follows:

(1) $$Mg+2H_{2}O\to Mg{\left(OH\right)}_{2}+H_{2}↑$$

(2) $$V\;\left(H_{2}\right)=m\left(Mg\right)\times M\left(H_{2}\right)\times \rho \left(H_{2}\right)/M\left(Mg\right)$$

### pH Measurement

The AZ31 magnesium alloy tubes were immersed in a PBS solution (pH 6.8). A portable pH meter (Sanxin Instrument, Shanghai, China) was used to measure the pH of the solution at different time points until the Mg‐alloy tubes were completely degraded.

### Cell Lines and Culture Conditions

Three types of tumor cell lines were used in this study: HepG2 cells were purchased from Cellcook Biotechnology Co., Ltd., Guangzhou, China; VX2 cells were obtained from OneShine Biotechnology Co., Ltd., Guangzhou, China; and MC38 cells were kindly provided by Professor Wei‐Jun Fan's research group at the Sun Yat‐sen University Cancer Center. All cell lines were cultured in a humidified incubator at 37 °C with 5% CO_2_ (Thermo Fisher Scientific, Waltham, MA, USA). HepG2 cells were cultured in Minimum Essential Medium (MEM, Gibco, Grand Island, NY, USA), while VX2 and MC38 cells were cultured in Dulbecco's Modified Eagle's Medium (DMEM, Gibco, Grand Island, NY, USA). Prior to experimental use, all cell lines were tested for mycoplasma contamination using the MycoBlue Mycoplasma Detector kit (Vazyme Biotech Co., Ltd., Nanjing, China) to ensure the absence of mycoplasma.

### CCK‐8 Assay

The Cell Counting Kit‐8 (CCK‐8) proliferation assay kit was purchased from Yishan Biotechnology, Shanghai, China. Briefly, cells were seeded in 96‐well plates at a density of 5000 cells per well or in 24‐well plates at a density of 20000 cells per well. After the designated treatment times, 10% CCK‐8 reagent in complete culture medium was added to each well. The plates were incubated at 37 °C in the dark for 2 h. The absorbance at 450 nm was then measured using a multifunctional microplate reader (BioTek Instruments, Winooski, VT, USA). Results were normalized to the 0 h absorbance readings.

### Detection of Mg^2+^ Concentration

Forty‐eight hours after treating the cells with the magnesium alloy tubes, the supernatant was collected. The concentration of Mg^2+^ was determined using the xylidyl blue method by measuring the absorbance at 500 nm. The Mg^2+^ concentration was calculated and expressed in mmol L^−1^.

### 5‐Ethynyl‐2′‐Deoxyuridine (EdU) Incorporation Assay

The EdU incorporation assay was performed using the Cell‐Light EdU Apollo In Vitro Kit (RiboBio, Guangzhou, China). Cells were seeded in six‐well plates, and culture medium containing 50 µm EdU was added to each well. The cells were incubated at 37 °C for 2 h. Following incubation, the cells were fixed with 4% paraformaldehyde (Biosharp, China) and permeabilized with 0.5% Triton X‐100 (Sigma‐Aldrich, St. Louis, USA). The cells were then stained with the pre‐prepared reaction solution at room temperature in the dark for 30 min and stained with 1× Hoechst 33 342. The staining was observed using an ECLIPSE Ti‐2 inverted fluorescence microscope (Nikon, Tokyo, Japan).

### Flow Cytometry

Flow cytometry for apoptosis detection was performed using the Annexin V‐AF647/PI Apoptosis Kit (Yishan Biotechnology, Shanghai, China). Briefly, the cells were resuspended in binding buffer. Then, 10 µL of Annexin V‐AF647 and 5 µL of propidium iodide (PI) were added to the cell suspension and incubated for 5 min. Flow cytometric analysis was conducted using a CytoFLEX flow cytometer (Beckman Coulter, Pasadena, CA, USA).

### Mitochondrial Membrane Potential Detection

The mitochondrial membrane potential was detected using a JC‐1 Mitochondrial Membrane Potential Assay Kit (Beyotime Biotechnology, Shanghai, China). Briefly, a pre‐prepared JC‐1 staining solution was added to a culture dish, and the cells were incubated at 37 °C for 20 min. After incubation, the supernatant was discarded, and the cells were washed twice with a pre‐cooled 1 × JC‐1 staining buffer. After adding 1 mL of complete cell culture medium, the cells were observed using an FV1000 confocal laser scanning microscope (Olympus).

### Immunofluorescence Assay

Cells were seeded at a density of 2 × 10^4^ cells per dish in glass‐bottom culture dishes. After fixation with 4% paraformaldehyde, the cells were permeabilized and blocked with a solution of 0.3% Triton X‐100 and 5% BSA (Huayun Biotechnology, Guangzhou, China) for 1 h at room temperature. The cells were then incubated overnight at 4 °C with γH2A.X antibody (1:400, #9178, Cell Signaling Technology, MA, USA). The next day, after a 1 h incubation at room temperature, the cells were incubated with Fluorescein Isothiocyanate(FITC)‐conjugated secondary antibody (1:500, A0568, Beyotime Biotechnology, Shanghai, China) for 1 h at room temperature. The cells were then stained with 4′,6‐diamidino‐2‐phenylindole (DAPI, Mikxlife, Shenzhen, China) for 3 min. After adding anti‐fade mounting medium (Reagan Biotechnology, Beijing, China), the cells were observed using an FV1000 confocal laser scanning microscope.

### Western Blot

Cells were lysed using a whole‐cell lysis assay kit (KeyGEN, Nanjing, China) containing phosphatase and protease inhibitors. Protein concentrations were determined using the BCA Protein Quantitation Assay Kit (KeyGEN, China). Equal amounts of total protein were then separated on a 4–20% SDS‐PAGE gel (Epizyme, China) and transferred onto polyvinylidene fluoride membranes. These membranes were incubated with primary antibodies specific to caspase‐3, cleaved caspase‐3, bcl‐2, BAX, γH2A.X, followed by secondary antibody incubation. Detection was performed using enhanced chemiluminescence (GBC Bio, China) on a ChemiDoc MP Imaging System (Bio‐Rad, USA). β‐actin was used as loading controls.

### In Vivo Experiments in Mice

In vivo experiments in mice were approved by the Committee on Animals of Sun Yat‐sen University (protocol code: L102012024030B). All animal experiments were performed in strict compliance with the National Institutes of Health Guide for the Care and Use of Laboratory Animals. Five‐week‐old female *C57BL/6J* mice were obtained from the Guangdong Medical Laboratory Animal Center and maintained under specific pathogen‐free conditions. Subcutaneous tumor xenografts were established using MC38 tumor cells. To enhance the tumor formation efficiency, an equal volume of Matrigel was added to the cell suspensions before injection. Each mouse was injected subcutaneously with 8.0 × 10^5^ MC38 cells into the right flank. The size of the tumor was monitored every 2 d, and subsequent experimental treatments were initiated when the tumors reached a diameter of 5–6 mm. Mice bearing tumors were randomly assigned to four groups, with five mice per group: group A: control group; group B: magnesium alloy tube group (Mg tube group); group C: radioactive ^125^I seed group (^125^I seed group); and group D: combined magnesium alloy tube and radioactive ^125^I seed group (combination group). Following anesthesia, the mice underwent direct visual implantation of a magnesium alloy tube and/or ^125^I seeds into the tumor center using a puncture needle. Because of the size constraints of the tumors, the magnesium alloy tube and ^125^I seeds were placed in parallel in the combination group. The mice in the control group underwent a sham operation involving needle puncture without actual implantation. The tumor size and body weight of the mice were monitored every 3 d. The hydrogen content within the tumors was measured weekly using a hydrogen microelectrode. After 3 weeks, the mice were euthanized, and the whole tumor tissue was excised. The tumor volume was calculated using Equation (3) as follows:

(3) $$V=a\times b^{2}/2\;$$

where *a* represents the tumor's long diameter and *b* represents the short diameter.

### In Vivo Experiments in Rabbits

In vivo experiments in rabbits were approved by the Experimental Animal Management and Use Committeeof Zhuhai People's Hospital (protocol code: 20240702‐001). This study utilized female New Zealand White rabbits weighing 2.0–2.5 kg, sourced from Guangdong Mingzhu Biotechnology Co., Ltd. VX2 tumor tissues were generously provided by Dr. Shiyu Zhang's research group at Guangzhou Medical University. Prior to surgery, the rabbits were fasted, anesthetized, and their fur was shaved. A 5 cm incision was made along the right subcostal margin. After accessing the abdominal cavity, the liver was gently palpated, and the right median lobe was carefully exteriorized. Two VX2 tumor tissue blocks were implanted into the liver using a syringe, and gelatin sponge strips were inserted into the puncture tracts to prevent dislodgement of the tumor blocks. The wound was disinfected with a povidone‐iodine solution, and the abdominal cavity was closed with sutures. Postoperatively, the rabbits received intramuscular injections of 400000 units of penicillin for three consecutive days. Ultrasound examinations were conducted weekly to monitor tumor growth, and experimental treatments were initiated ≈3 weeks post‐implantation.

Three weeks after tumor implantation, the rabbits underwent CT imaging (GE Healthcare, Chicago, Illinois, USA). Scans were performed in the lateral or supine position to assess tumor size, ^125^I seed distribution, and complete preoperative planning using the treatment planning system (TPS). The TPS plan was developed using a brachytherapy treatment planning system (Astro Technology, Beijing, China). Preoperative CT was used to delineate the GTV. The prescribed radiation dose (PD) was 120 Gy. Simulations were performed by placing 0.8 mCi 6711‐type ^125^I seeds within the tumor and adjusting the number and arrangement of seeds to display the isodose curves. The preoperative TPS plan was completed with the assistance of a radiation physicist with over five years of experience.

After completing the TPS plan, the materials were implanted into the rabbits. After opening the abdominal cavity, the tumor was exteriorized gently. ^125^I seeds or seed strands were implanted by inserting a puncture needle into the tumor, retracting the inner needle, and placing the seeds into the needle shaft. The seeds were gently pushed into the tumor tissue using a needle core and the puncture needle was withdrawn. Gelatin sponge strips were quickly inserted into the puncture tract to prevent tumor cell seeding and metastasis. The abdomen was then sutured and closed, and the rabbits received intramuscular injections of 400000 units of penicillin for three consecutive days postoperatively.

Blood samples were collected from rabbits at designated time points after implanting the ^125^I seeds/seed strands. The collected blood was analyzed for complete blood counts, ALT, AST, CREA, UREA, and magnesium ion levels. This analysis was conducted with assistance from the Guangzhou Huayin Medical Laboratory Center, China.

### Hematoxylin‐Eosin (H&E) Staining

Histological changes in various organs (heart, liver, spleen, lung, and kidney) were assessed. The organs were harvested and fixed in 4% paraformaldehyde. Following fixation, the tissues were embedded in paraffin, sectioned, and stained with H&E. The tissue sections were scanned using a KF‐PRO‐020 Jiangfeng automatic slide scanner (KFBio, Ningbo, China).

### Immunohistochemistry (IHC)

Following paraffin embedding and sectioning, deparaffinization, and hydration, the sections underwent inactivation with 3% H2O2 for 10 min, followed by PBS washes. Antigen retrieval was performed using EDTA solution heated in a microwave for 5 min. The sections were blocked with 5% BSA at 37 °C for 30 min, incubated with primary antibodies at 37 °C for 2 h, washed, then incubated with secondary antibodies at 37 °C for 30 min and washed again. DAB was applied for color development, monitored microscopically, then washed and dried. Hematoxylin staining was done for 40 s, followed by bluing solution, washing, mounting, and microscopic observation.

### Statistical Analysis

Statistical analysis was conducted using SPSS version 23.0 (IBM, Armonk, NY, USA). Data conforming to a normal distribution were presented as mean ± standard deviation and analyzed for homogeneity of variance using the F‐test, followed by the Student's t‐test for differences between groups. For non‐normally distributed data, the median ± interquartile range was used, and the Wilcoxon test was performed for difference analysis. GraphPad Prism 8 (GraphPad Software, San Diego, CA, USA) was used for statistical plotting. All tests were two‐sided, with *p* < 0.05 considered statistically significant, indicated as * for *p* < 0.05, ** for *p* < 0.01, *** for *p* < 0.001, and ns (no significance) for *p* ≥ 0.05.

## Conflict of Interest

The authors declare no conflict of interest.

## Supporting information



Supporting Information

## Data Availability

The data that support the findings of this study are available from the corresponding author upon reasonable request.
