# Supplementary material for: Hydrogen‐Generating Magnesium Alloy Seed Strand Sensitizes Solid Tumors to Iodine‐125 Brachytherapy
Source: Adv Sci (Weinh). 2024 Dec 10;12(5):2412263. doi: 10.1002/advs.202412263 (PMC11792047; doi:10.1002/advs.202412263)
Supplement: Supplementary file 1 — Supporting Information [file ADVS-12-2412263-s001.docx]

Supporting Information

Hydrogen-Generating Magnesium Alloy Seed Strand Sensitizes Solid Tumors to Iodine-125 Brachytherapy

*Pan Hu, Letao Lin, Guanyu Chen, Dengyao Liu, Huanqing Guo, Meigui Xiao, Zhihui Zhong, Guang Yang, Bingchen Xu, Dongcun Huang, Sheng Peng, Yong Li, Yanling Zhang*, Tao Huang*, Fujun Zhang**


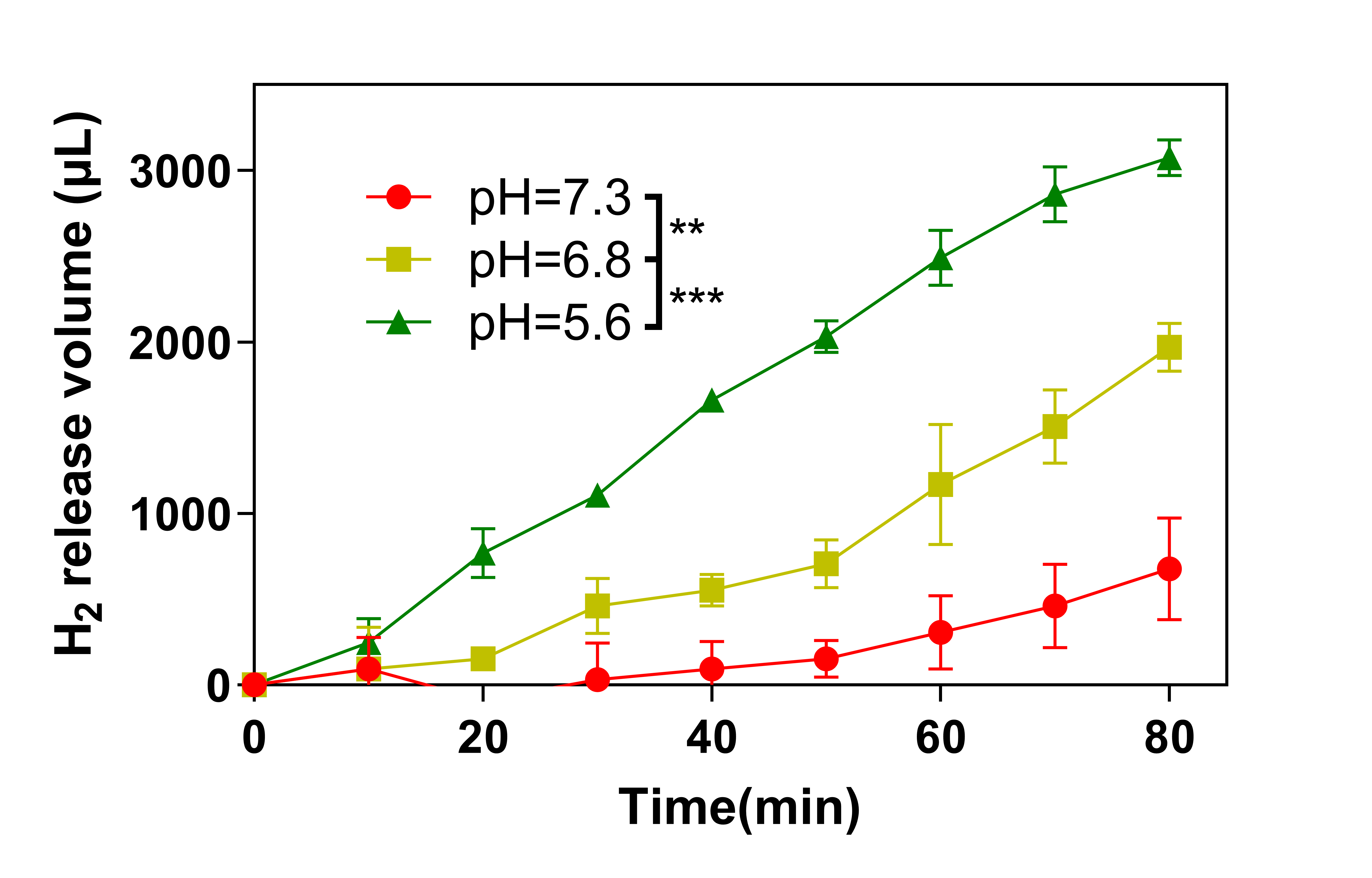


**Figure S1.** Hydrogen volume produced from AZ31 magnesium alloy tubes calculated under different pH values.


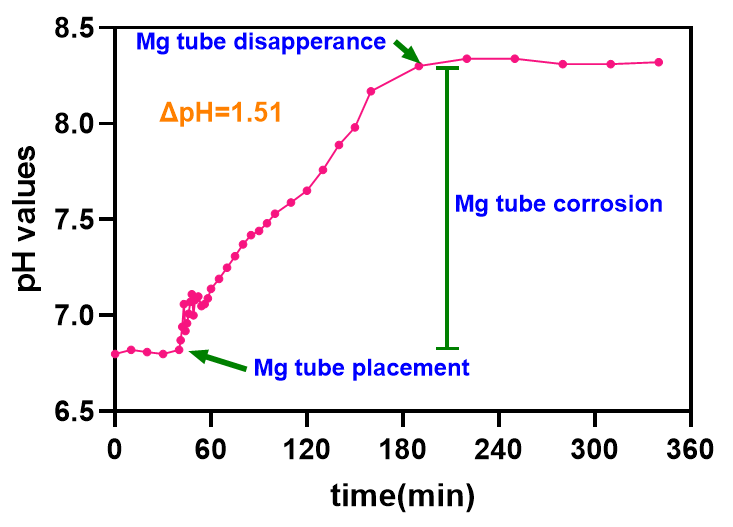


**Figure S2.** The effect of AZ31 magnesium alloy tubes on the pH of the solution (pH=6.8).

**Table S1.** The concentration of Mg^2+^ in the cell culture medium with AZ31 magnesium alloy tubes

| **Sample** | **Mg^2+^ concentration [mmol/L]** |
| --- | --- |
| 1 | 5.09 |
| 2 | 5.05 |
| 3 | 5.11 |
| Mean±SD | 5.08±0.02 |


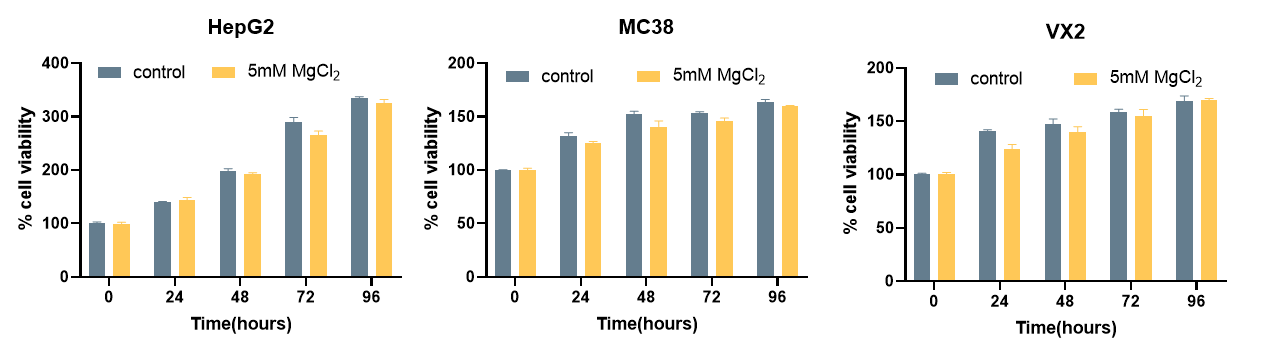


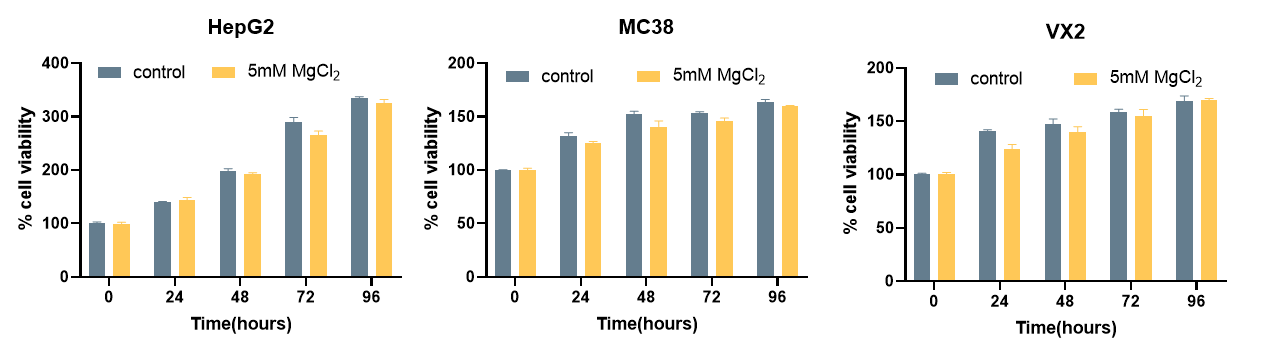


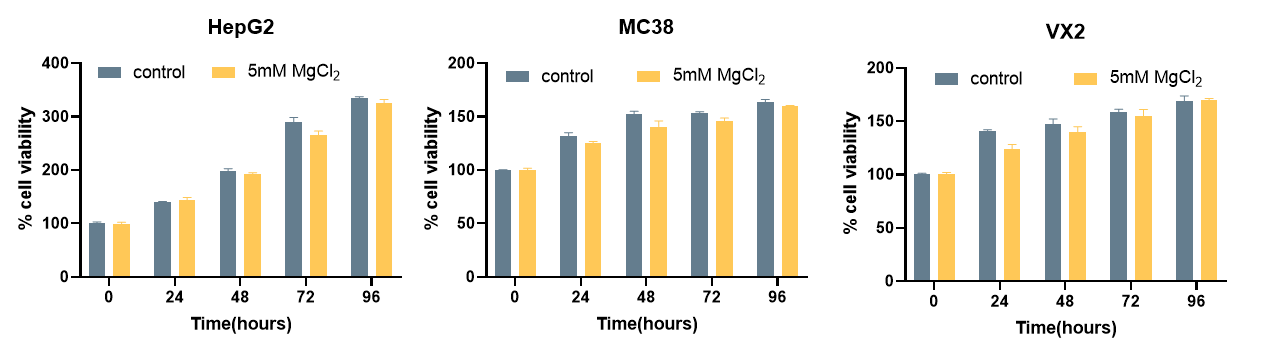


**Figure S3.** CCK-8 assay demonstrates that 5 mM Mg^2+^ has no effect on the proliferation capability of three types of tumor cells.


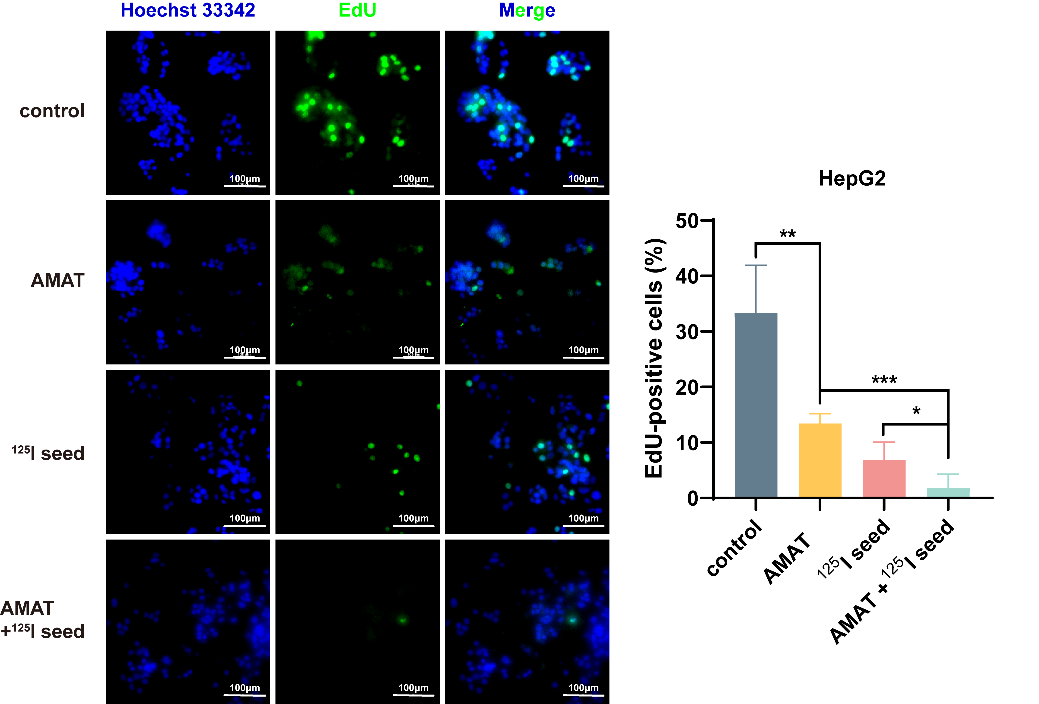


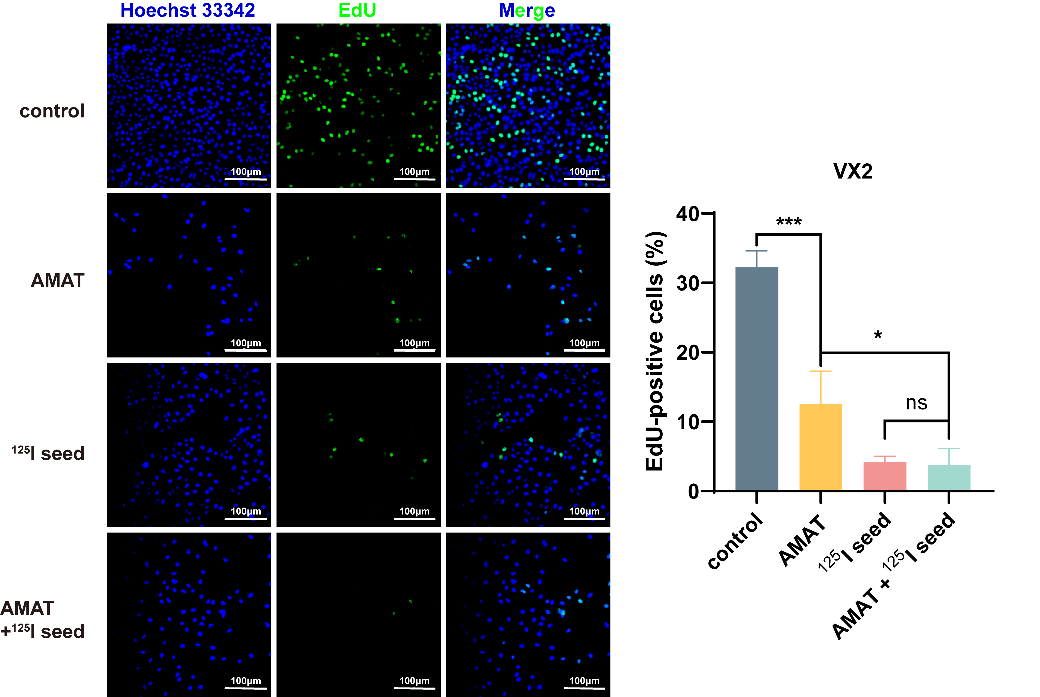


**Figure S4.** EdU incorporation experiments confirm that hydrogen production from magnesium alloy tubes enhances the inhibitory effect of ^125^I seeds on the proliferation ability of HepG2 and VX2 cells.

**Figure S5.** CCK-8 experiments demonstrate that hydrogen production from magnesium alloy tubes enhances the inhibitory effect of ^125^I seeds on the proliferation capability of HepG2 and VX2 cell lines.


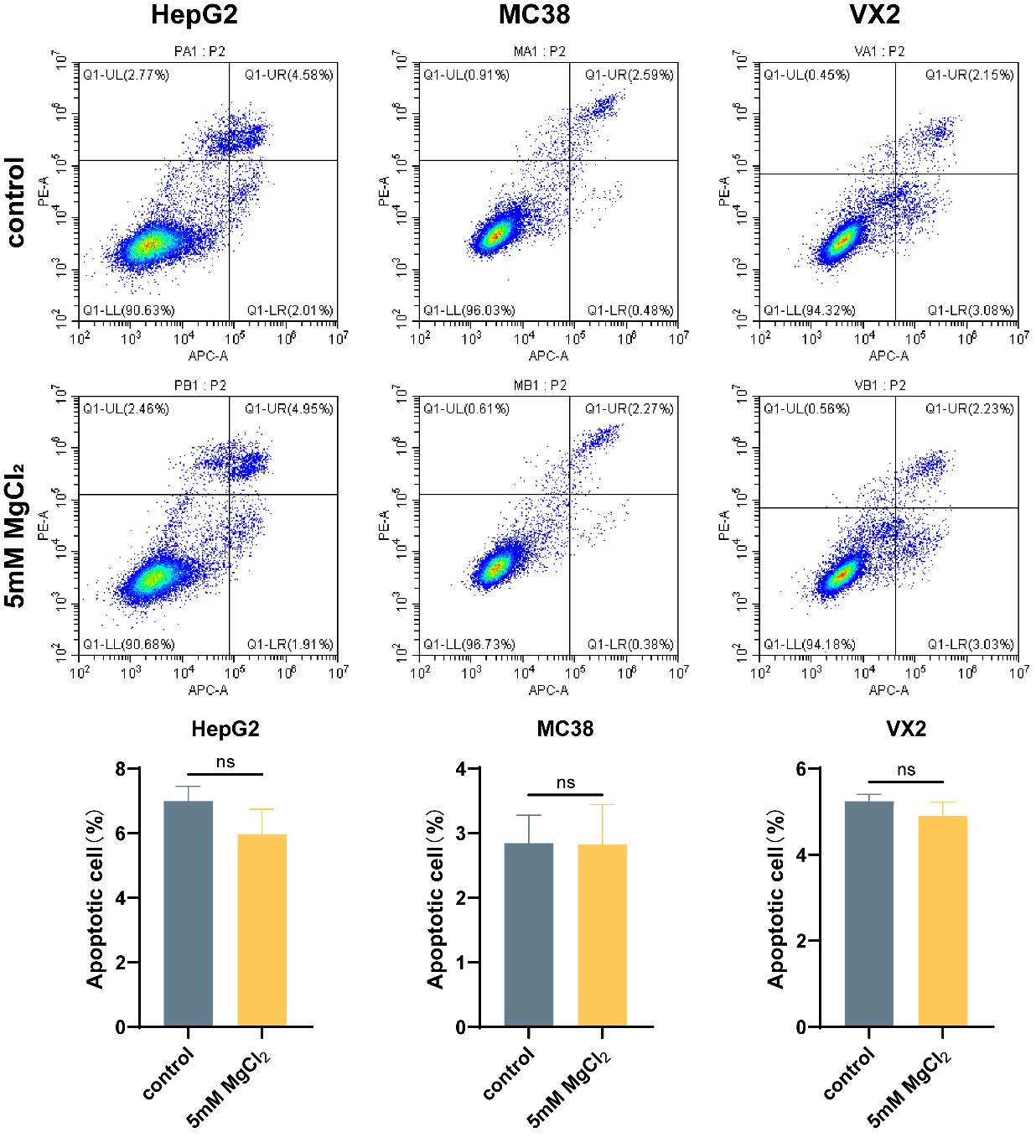


**Figure S6.** Flow cytometry assay demonstrates that 5 mM Mg^2+^ has no effect on the apoptosis rate of three types of tumor cells.


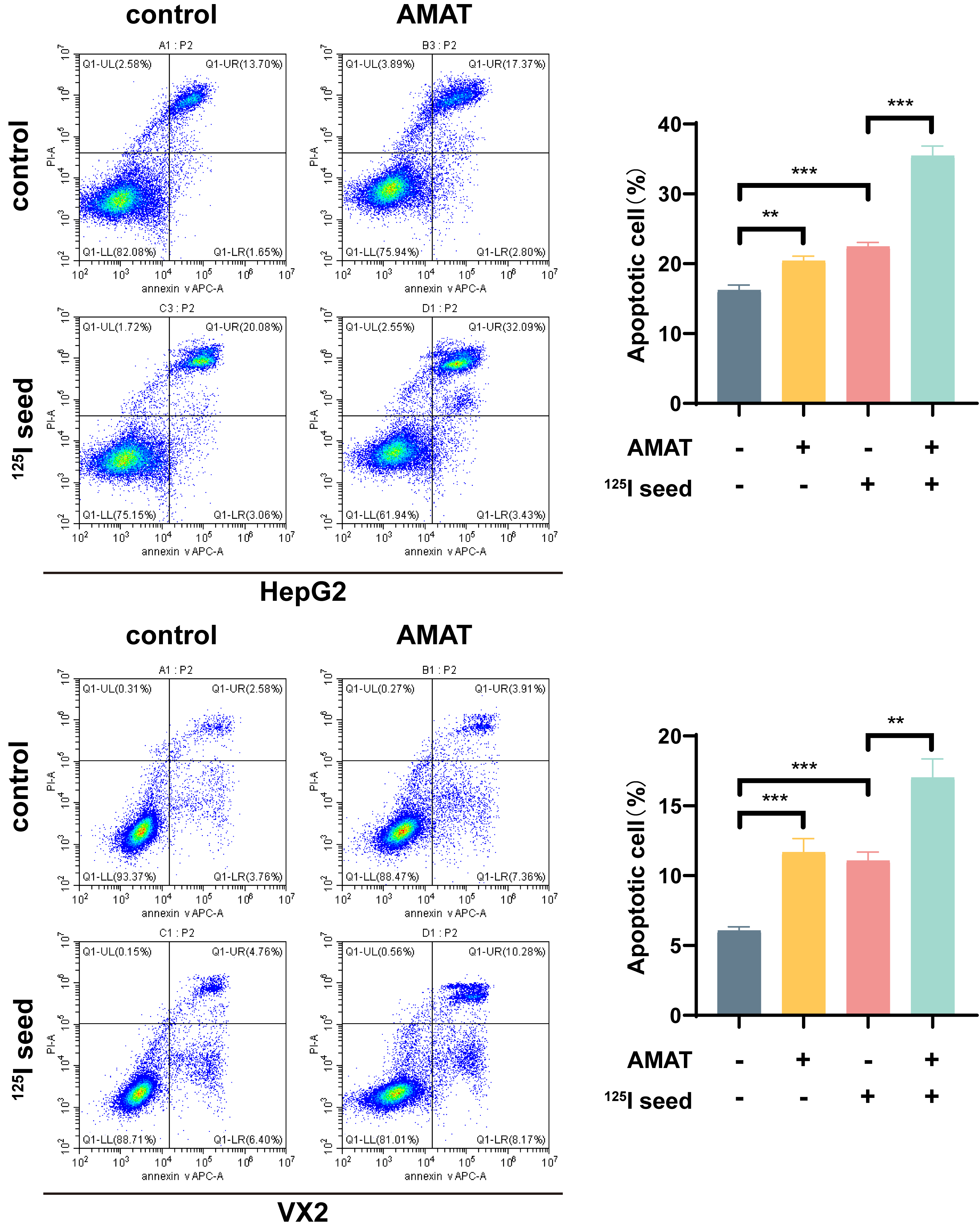


**Figure S7.** Flow cytometry confirms that hydrogen production from magnesium alloy tubes enhances the promoting effect of ^125^I seeds on the apoptosis rate of HepG2 and VX2 cells.

**
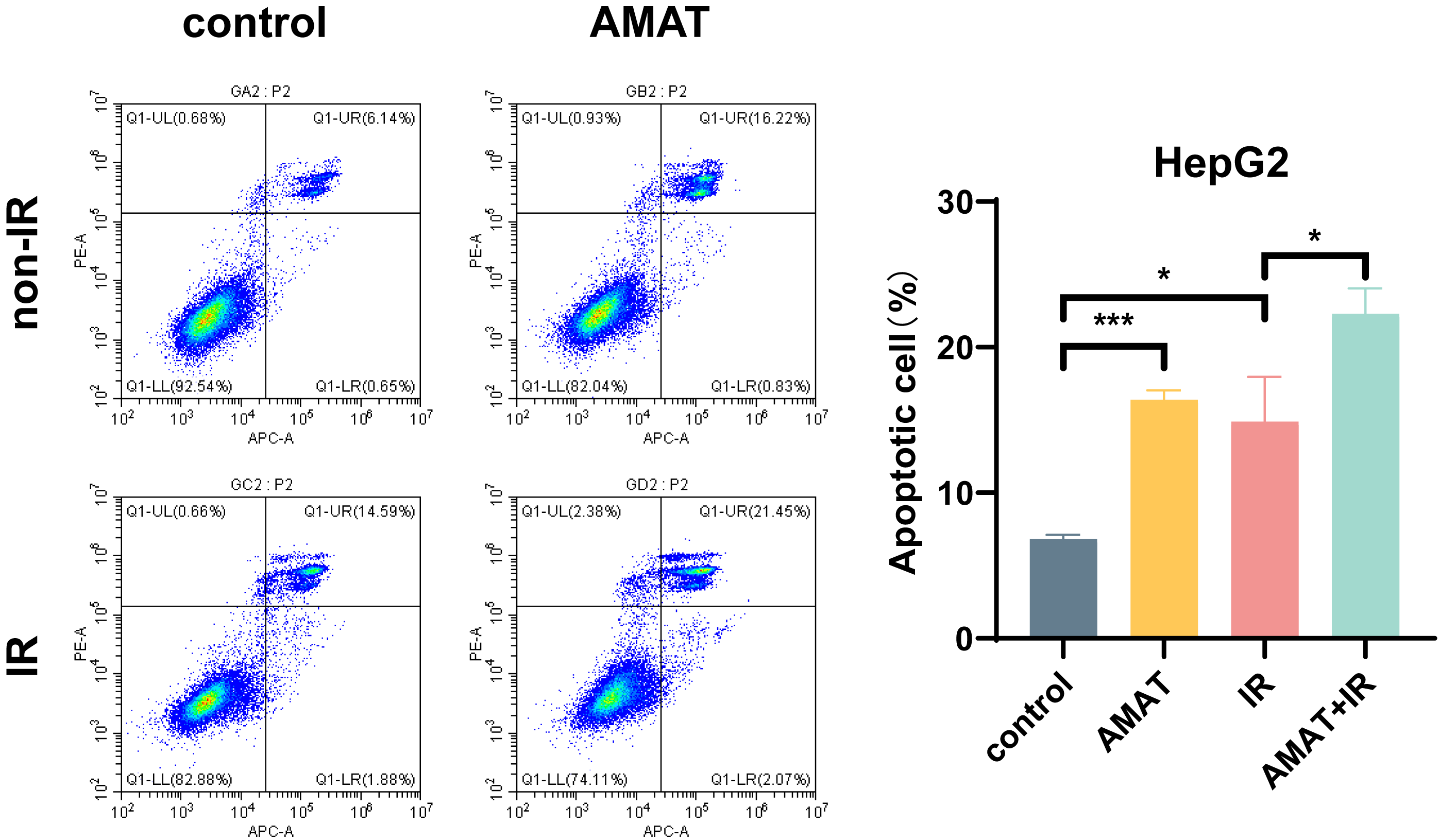
**

**
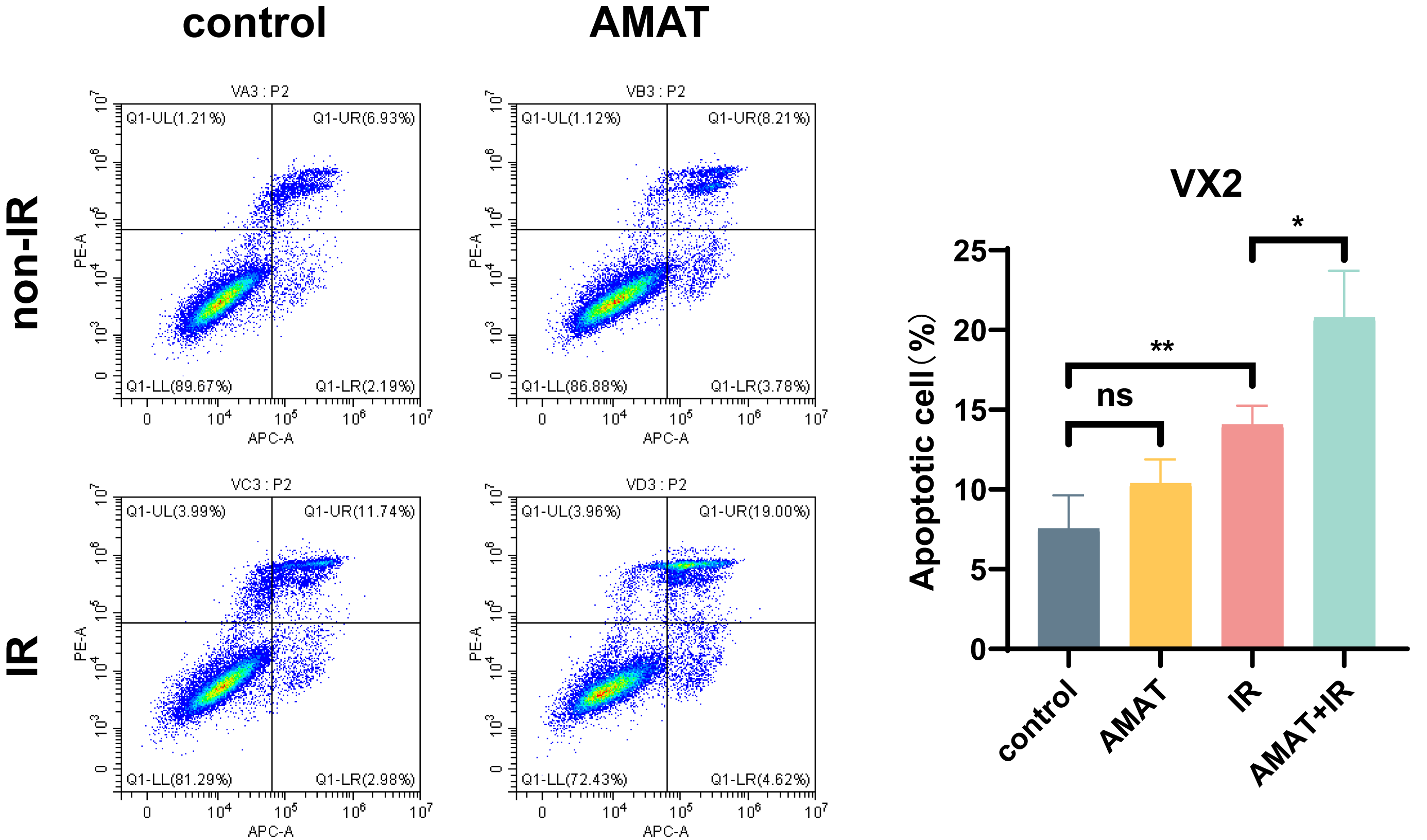
**

**Figure S8.** Flow cytometry confirms that hydrogen production from magnesium alloy tubes enhances the promoting effect of external beam radiation therapy on the apoptosis rate of HepG2 and VX2 cells.

**
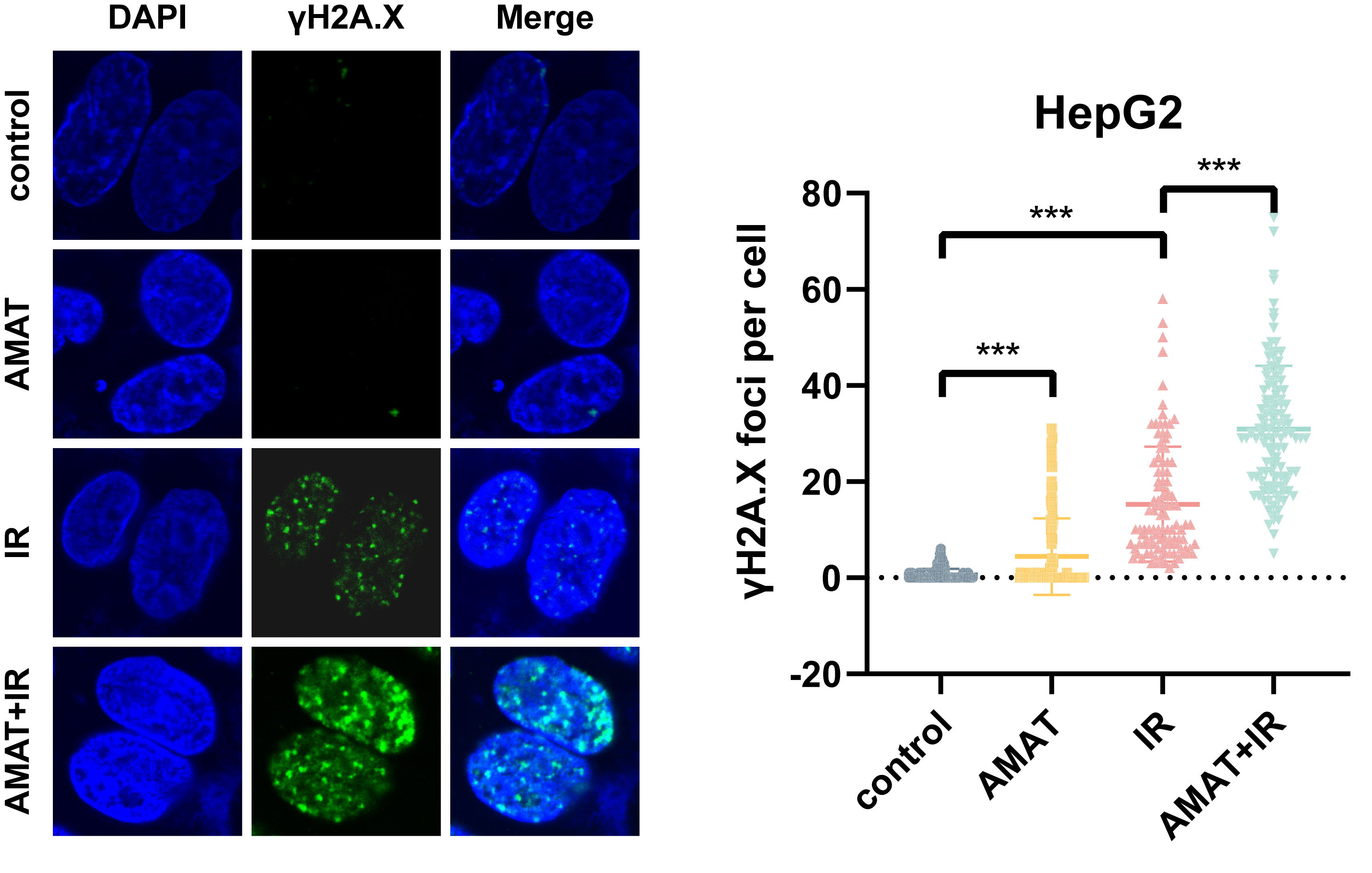
**

**
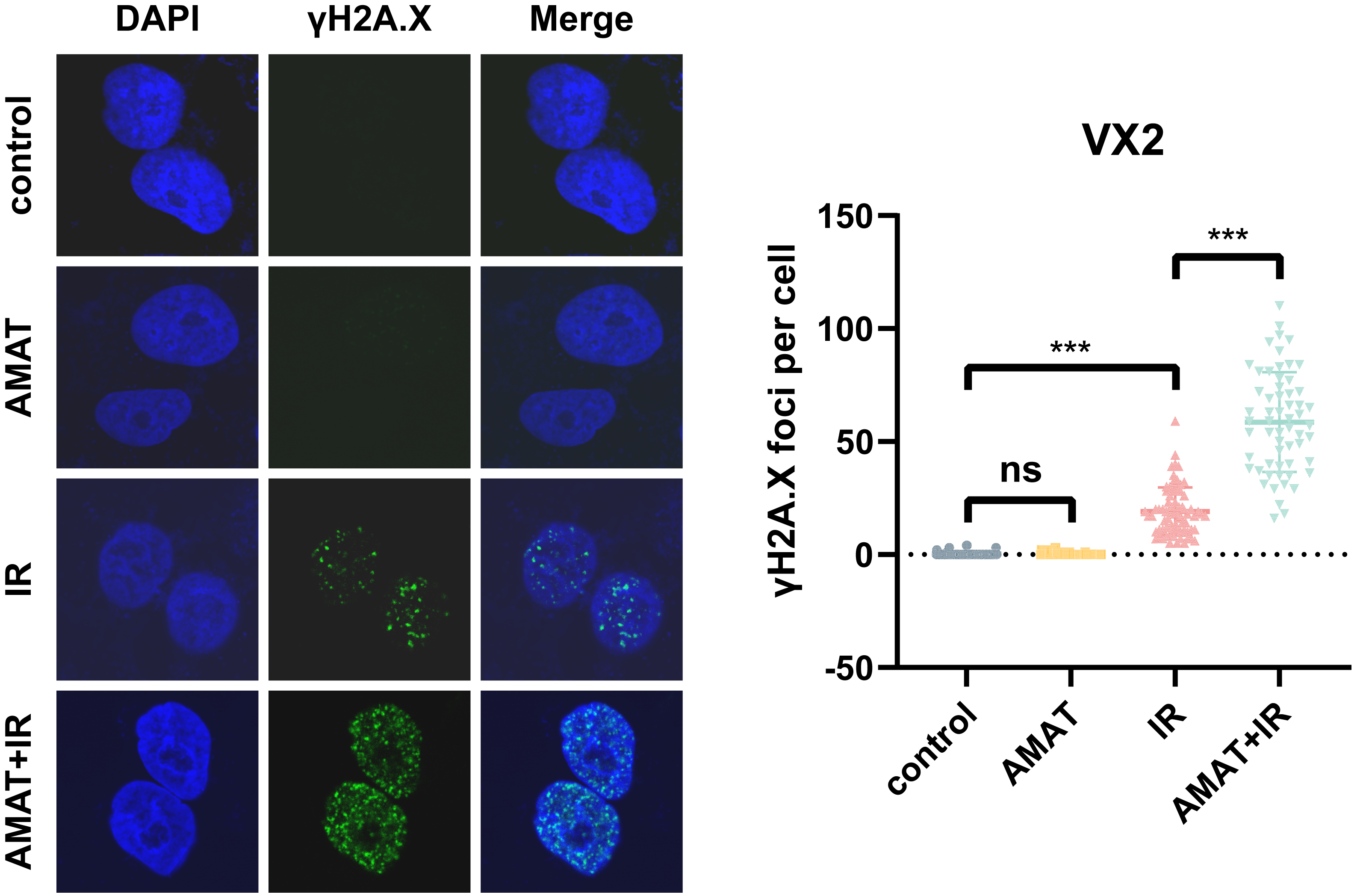
**

**Figure S9.** Immunofluorescence assay confirms that hydrogen production from magnesium alloy tubes promotes the DNA damage induced by external beam radiation therapy in HepG2 and VX2 cells.


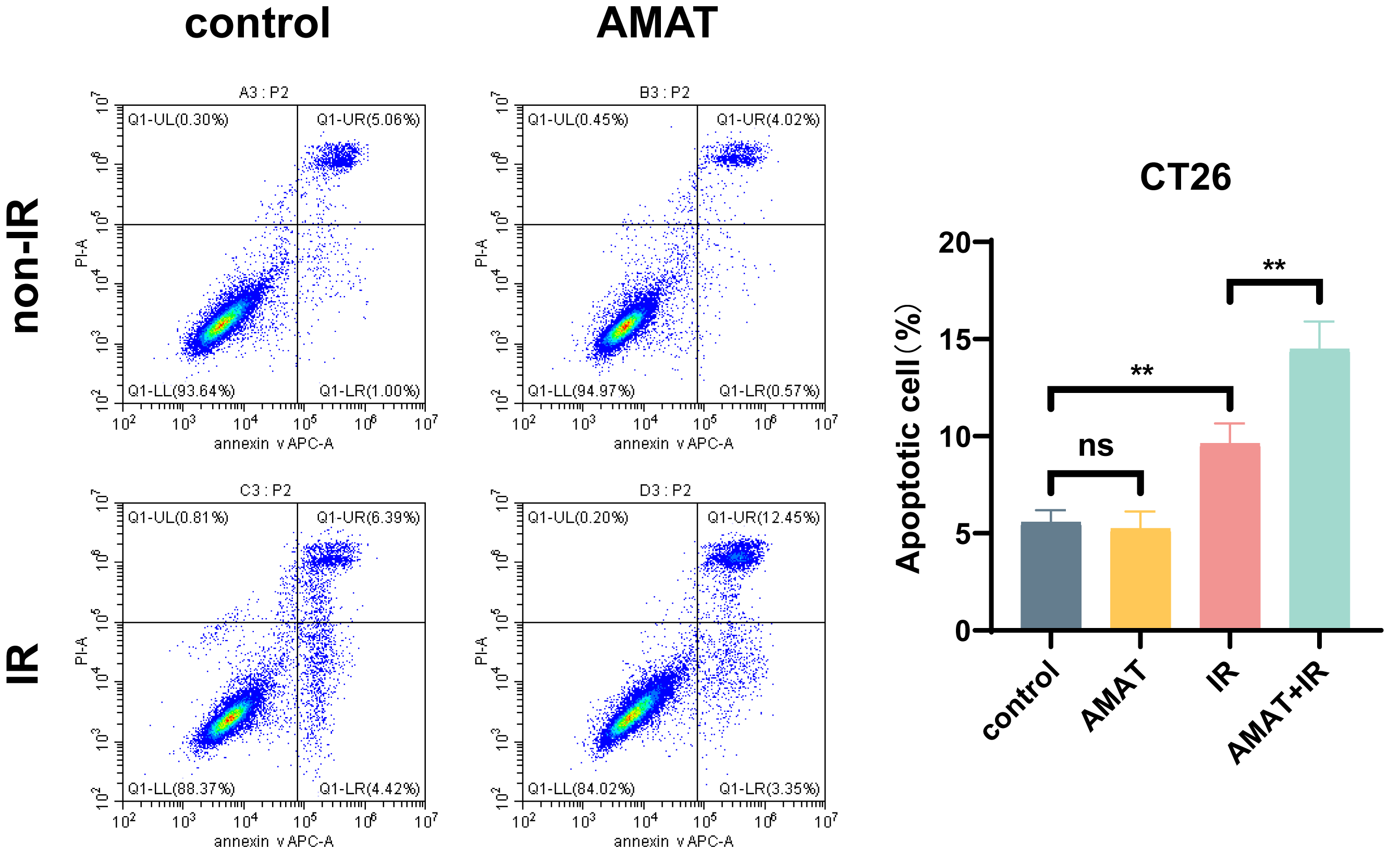


**Figure S10.** Flow cytometry confirms that hydrogen production from magnesium alloy tubes enhances the promoting effect of external beam radiation therapy on the apoptosis rate of CT26 cells.

**
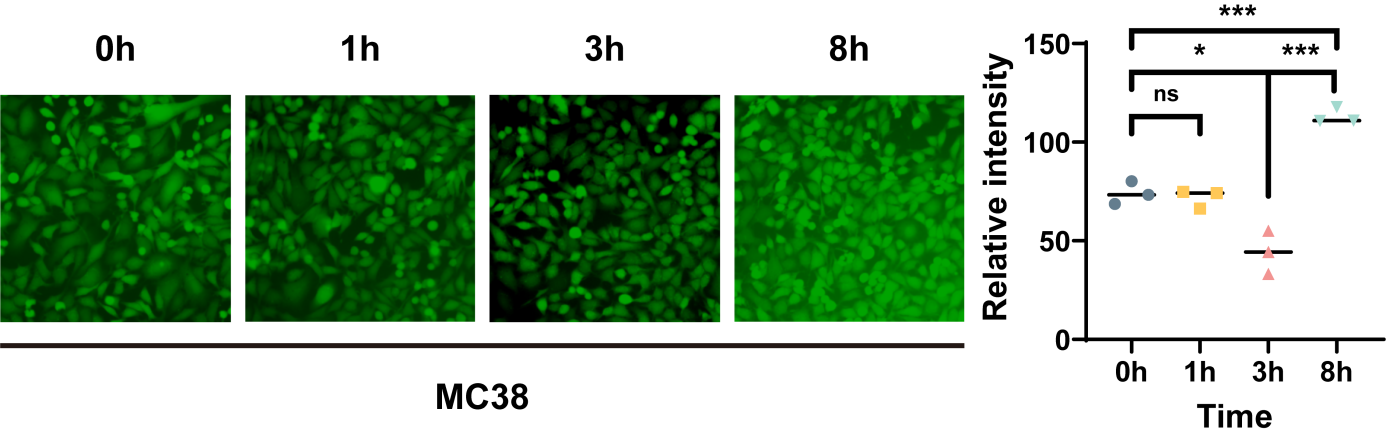
**

**Figure S11.** ROS levels in MC38 cells at different time points following AZ31 magnesium alloy tube treatment.

**
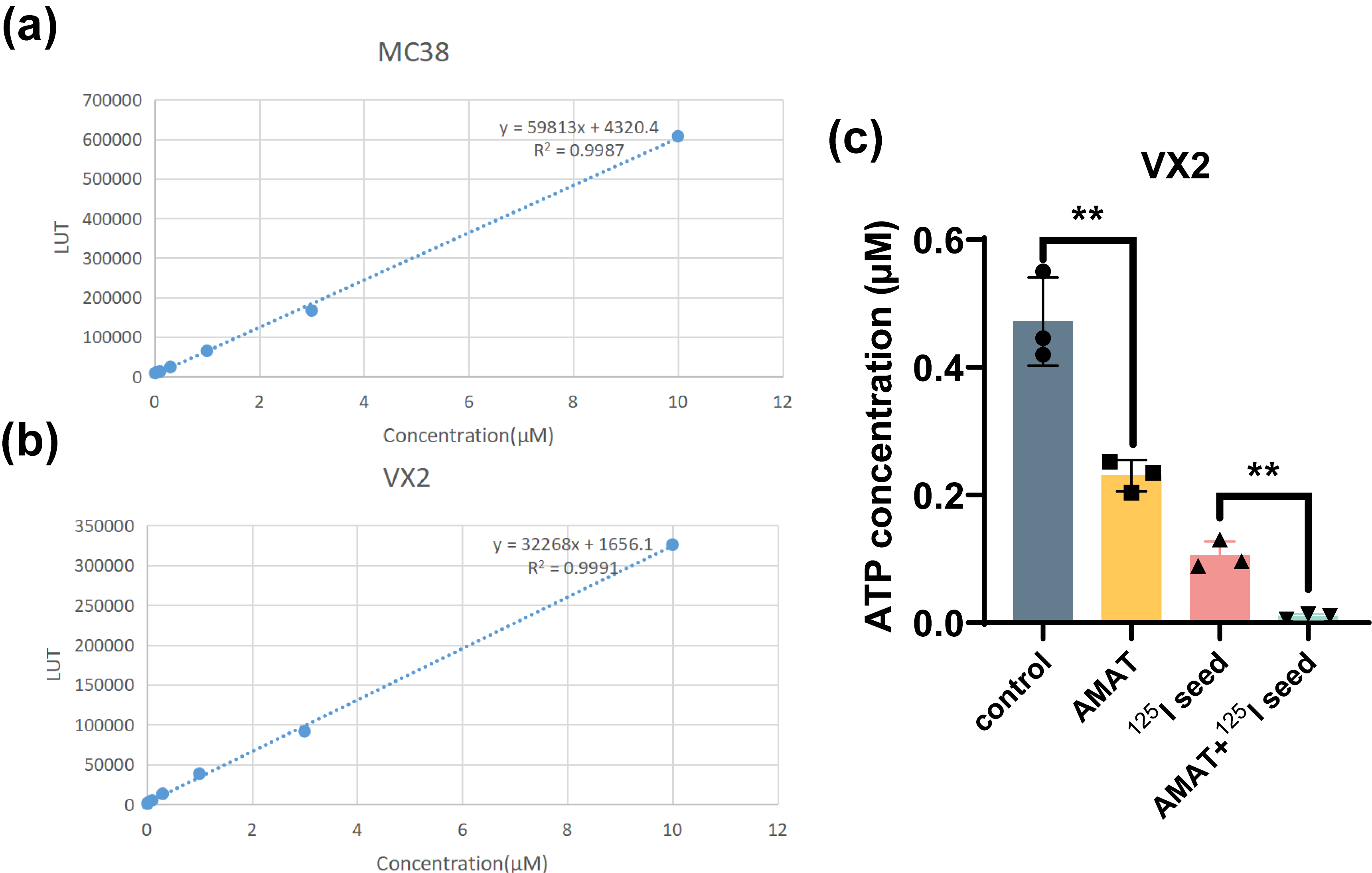
**

**Figure S12.** Intracellular ATP levels following treatment with AZ31 magnesium alloy tubes and ^125^I seeds. a) Standard curve for ATP quantification in MC38 cells. b) Standard curve for ATP quantification in VX2 cells. c) Intracellular ATP levels of VX2 cells following treatment with AZ31 magnesium alloy tubes and ^125^I seeds.


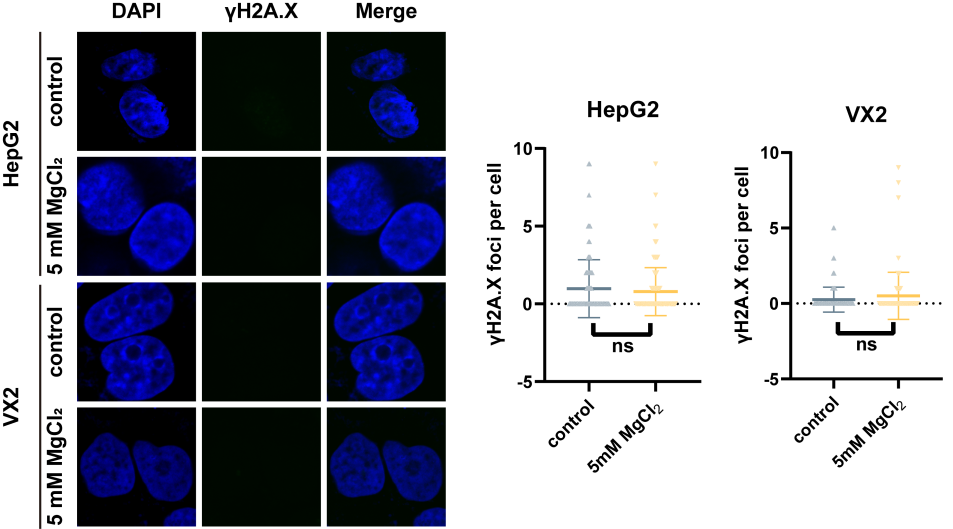


**Figure S13.** Immunofluorescence assay demonstrates that 5 mM Mg^2+^ has no effect on the DNA damage of HepG2 and VX2 cells.


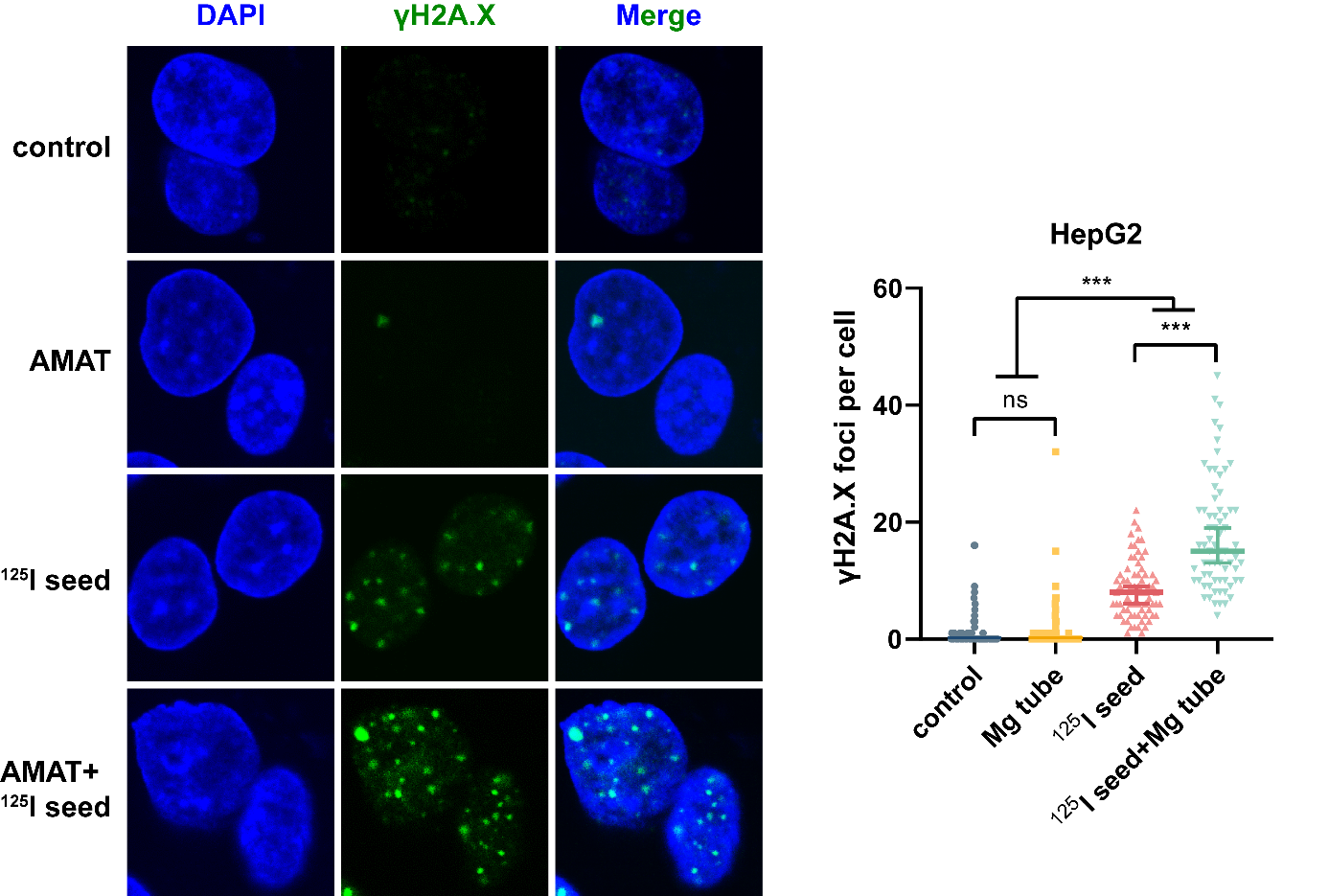


**Figure S14.** Immunofluorescence assay confirms that hydrogen production from magnesium alloy tubes promotes the DNA damage induced by ^125^I seeds in HepG2 cells.


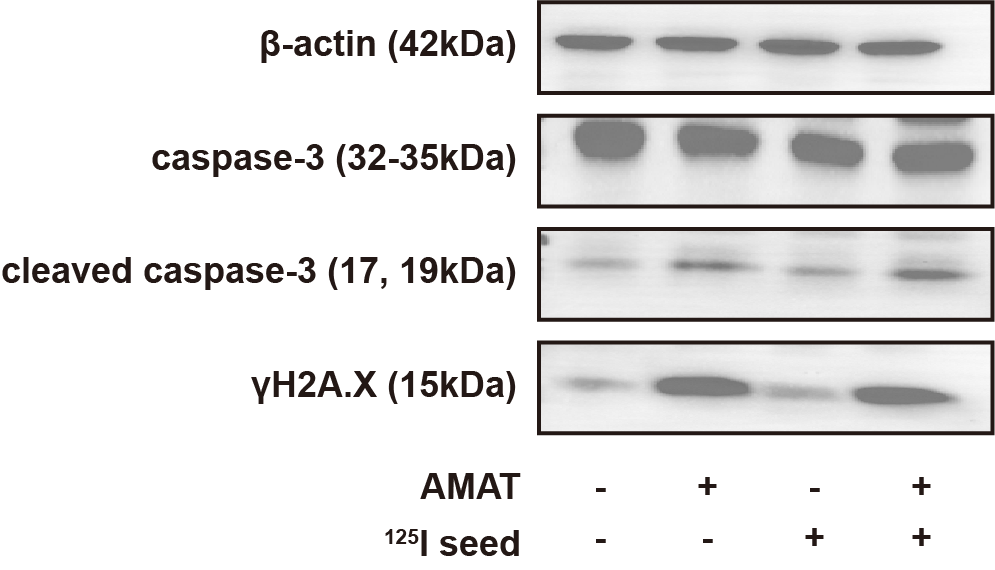


**Figure S15.** The combined effect of hydrogen production from magnesium alloy tubes and radioactive ^125^I seeds on DNA damage and apoptosis markers in MC38 cells.


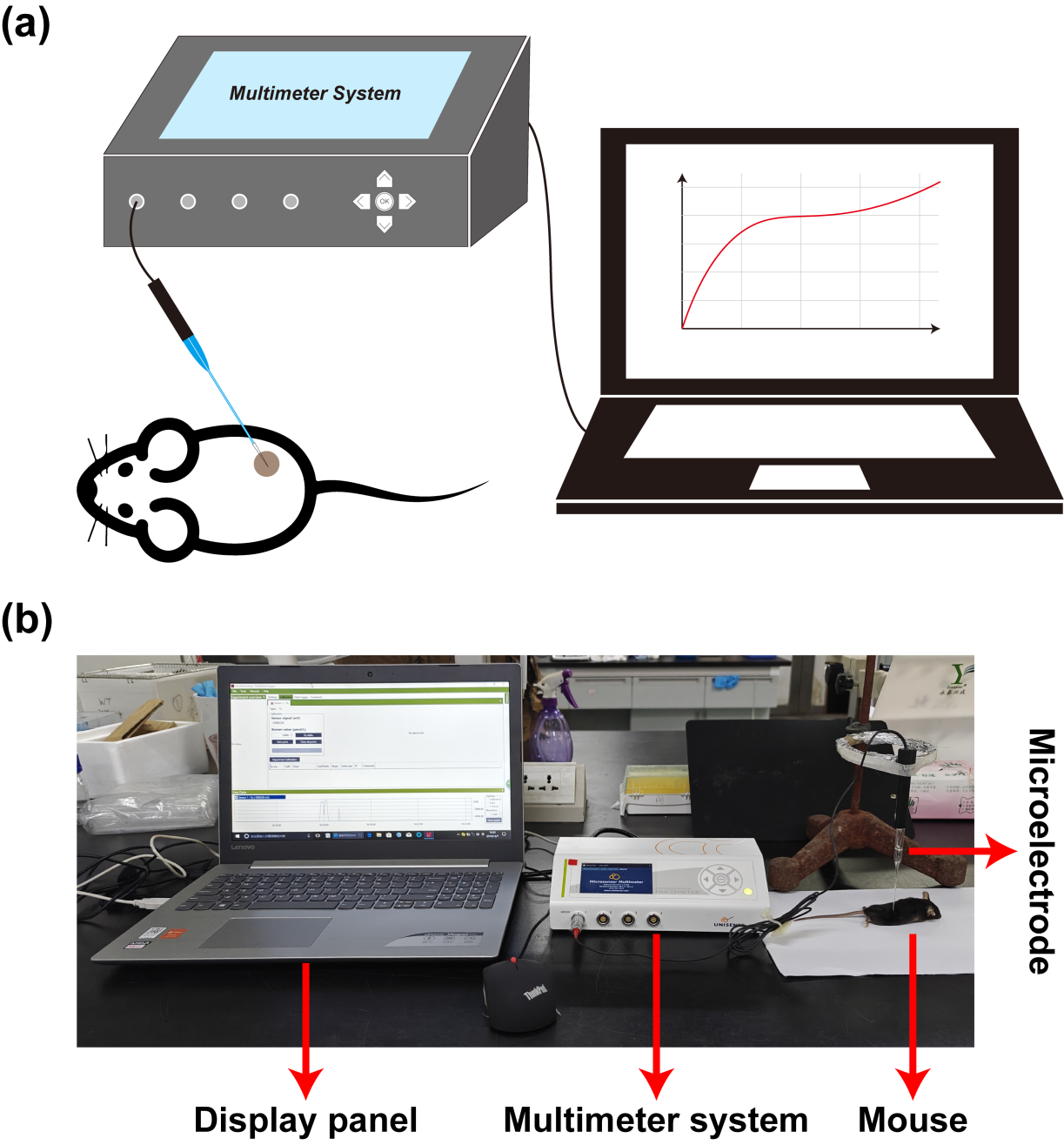


**Figure S16.** Using microelectrodes for hydrogen measurement within mouse tumors. a) Schematic diagram; b) Physical image


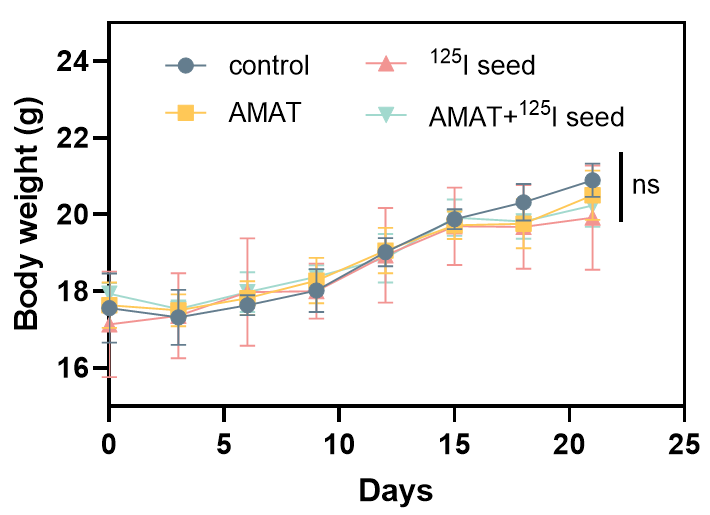


**Figure S17.** Mouse weight statistical chart.


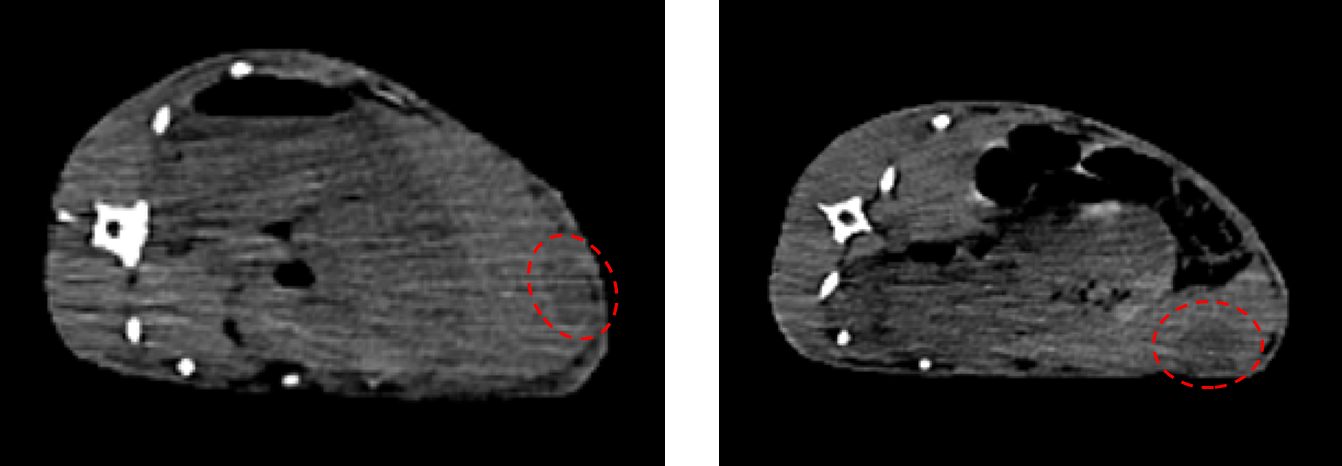


**Figure S18.** CT scan taken one day before implantation of magnesium alloy seed strands shows the formation of circular tumors.


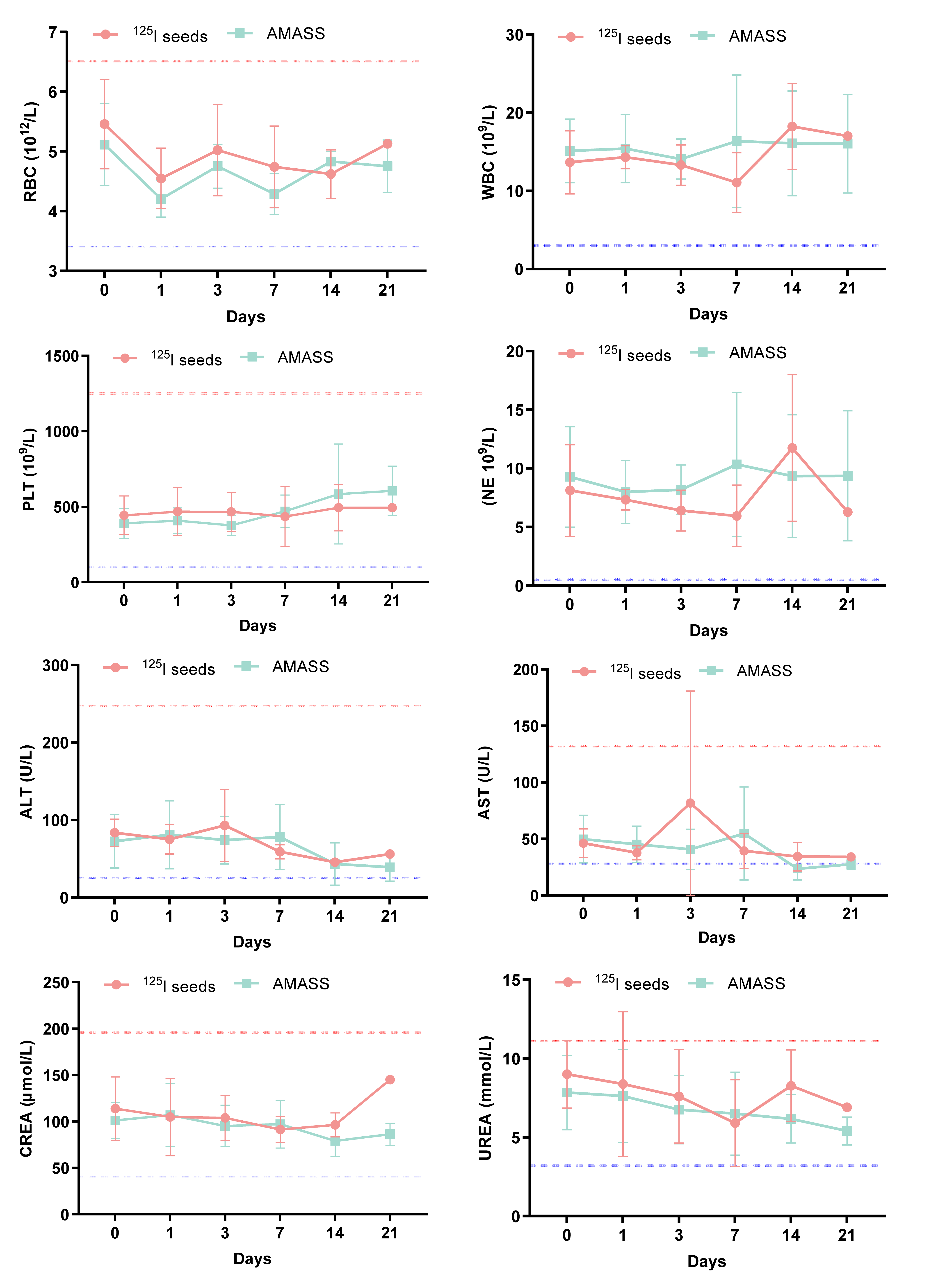


**Figure S19.** Changes in blood routine, liver function and renal function after implantation of seeds (strands) in New Zealand rabbit liver tumor model.


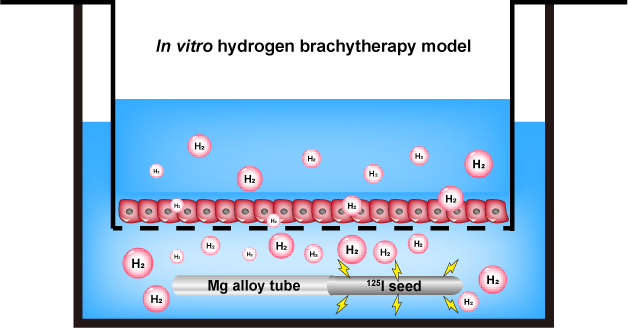


**Figure S20.** *In vitro* hydrogen brachytherapy model.


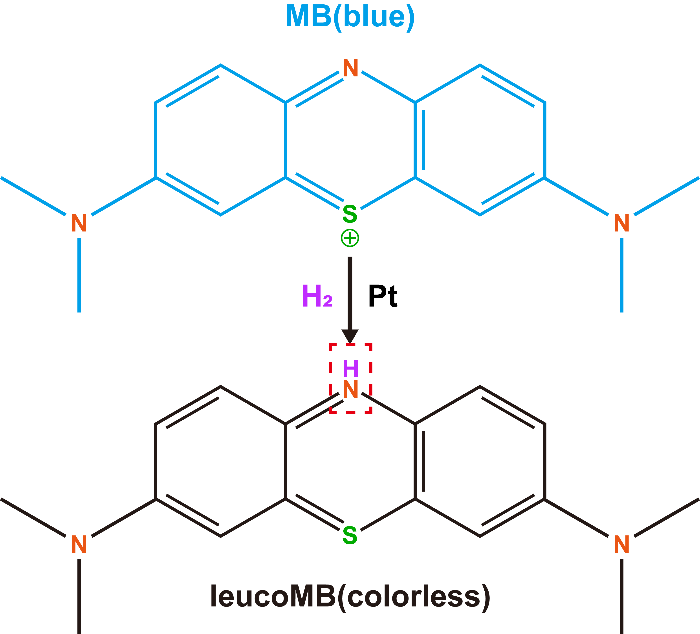


**Figure S21.** The schematic illustration of the methylene blue method for hydrogen detection.
